# The *FLNA* Gene in Tumour‐Educated Platelets Can Be Utilised to Identify High‐Risk Populations for NSCLCs

**DOI:** 10.1111/jcmm.70544

**Published:** 2025-04-10

**Authors:** Ruiling Zu, Hanxiao Ren, Xing Yin, Xingmei Zhang, Lubei Rao, Pingyao Xu, Dongsheng Wang, Yuping Li, Huaichao Luo

**Affiliations:** ^1^ Department of Clinical Laboratory, Sichuan Clinical Research Center for Cancer Sichuan Cancer Hospital and Institute, Sichuan Cancer Center, Affiliated Cancer Hospital of the University of Electronic Science and Technology of China Chengdu China; ^2^ College of Medical Technology, Chengdu University of Traditional Chinese Medicine Chengdu China

**Keywords:** biomarker, cancer screening, *FLNA*, liquid biopsy, NSCLC, tumour‐educated platelets

## Abstract

Selective screening of the population based on NSCLC risk is an effective technique for minimising overdiagnosis and overtreatment. Platelets and the components can be used as liquid‐biopsy markers, potentially assessing the risk of NSCLC, which are easily deployed in clinical applications. Platelet RNA sequencing datasets were analysed to identify specific genes derived from NSCLC patients and healthy donors. Then, expressions of the selected gene were validated in a clinical trial. Not only the availability of the specific gene in differentiating NSCLC patients from healthy donors but also from patients with benign nodules was estimated respectively. Finally, the values of the specific TEP‐gene in metastasis and survival prognosis were also evaluated. *FLNA* was selected based on the GSE datasets, of which mRNA expression levels were higher in platelets from NSCLC patients than in healthy donors and also higher than in benign patients. To discriminate the malignant patients from the healthy individuals, *FLNA* got an AUC for the ROC curve of 0.716. When discriminating from the benign individuals, *FLNA* got an AUC of 0.705. In addition, an AUC of 0.595 was found when the metastatic group was distinguished from the non‐metastatic group using the relative quantitative results of *FLNA*, and it seemed that the high‐*FLNA*‐expression group had a poorer long‐term survival rate than the low‐expression group. These findings suggested that high expression of *FLNA* in TEPs may indicate the incidence and metastasis of NSCLC and serve as a biomarker for high‐risk estimation for NSCLC.

## 1 Introduction

For a very long time, lung cancer has been the first malignant tumour to kill a person in both sexes as well as the first instance in men. Lung cancer became the first malignant tumour in terms of incidence and death parity for both men and women in 2022, accounting for 17.5% of female malignant tumours, overtaking breast cancer (15.6%) [1]. Non‐small cell lung cancer (NSCLC) is one of the main types of lung cancer, comprising approximately 85%, mainly including lung adenocarcinoma (LUAD) and lung squamous cell carcinoma (LUSC) [2]. The 5‐year survival rate for lung cancer is a pitiful 20%. However, early asymptomatic detection of lung cancer through screening can enable treatment before advanced‐stage progression and lower mortality [3, 4].

The results of a sizable population‐based investigation verify that low‐dose chest CT (LDCT) screening lowers the death rate associated with lung cancer while improving early detection and decreasing the proportion of patients who proceed to the advanced stages of the disease [5]. In high‐risk individuals, LDCT screening can dramatically lower the death rate from lung cancer. The results of LDCT screening in Chinese communities for individuals at high risk of lung cancer revealed a positive rate of lung nodules as high as 22.9% (804/3512); of these, 6.34% (51/804) of patients had malignant nodules, and 1.5% (51/3512) of patients had lung cancer detected [6]. There are associated concerns with LDCT screening. With false‐positive results leading to needless invasive testing and financial strain, LDCT screening may involve over‐screening for some inactive diseases. LDCT screening also carries some dangers, such as expense, unintentional injury, radiation exposure, and needless tests and procedures.

As a result, risk factor evaluation for lung cancer can be used to meet the goal of adequately screening those who are at risk without over‐screening. In clinical practice, serum tumour markers are also frequently employed for tumour screening since they are a simple, easy‐to‐detect, and standardised liquid biopsy technique. Carcinoembryonic antigen (CEA), neuron‐specific enolase (NSE), cytokeratin 19 fragment 21–1 (CYFRA21‐1), gastrin‐releasing peptide precursor (Pro‐GRP), and squamous epithelial cell carcinoma antigen (SCC) are currently the serum markers that are frequently employed in the clinic to detect lung cancer. However, the sensitivity and specificity of the aforementioned indicators for early‐stage lung cancer are suboptimal [7]. Tumour‐educated platelets are a novel liquid biopsy technique that has been coming into the focus of researchers in recent years.

In addition to changing the proteins and nucleic acids within the platelets, tumour cells activate the platelets and encourage the bone marrow to create more platelets [8]. As a result, platelets become TEPs in the tumour environment, and the nucleic acids and proteins within the platelets may be affiliated with tumour cells [9, 10]. Peripheral blood contains a large number of easily extracted platelets, and the tumour‐specific nucleic acids inside them may be useful in determining the risk of tumour development. Consequently, a novel form of low‐invasive, radiation‐free liquid biopsy has emerged that uses peripheral blood extraction to separate platelets and identify platelet‐specific nucleic acids [11, 12]. A prediction algorithm was developed using the differential expression of TEP mRNA profiles, which could separate healthy individuals from NSCLC patients with a high accuracy rate [13, 14]. These studies suggest that platelet RNA appears to be a potential biomarker for the diagnosis, prognosis, prediction, or monitoring of NSCLC.

In this study, we characterised the gene expression of platelets in NSCLC by analysing the platelet high‐throughput sequencing data, and *FLNA* was selected as a specific mRNA of TEPs. Our results indicated that the expression of *FLNA* mRNA was significantly increased in platelets from NSCLC patients as compared to healthy donors and patients with benign lung nodules. The results revealed the potential value of *FLNA* in distinguishing NSCLC patients from healthy donors and patients with benign lung nodules. Furthermore, we discovered a correlation between the *FLNA* of TEPs and the metastasis and survival prognosis of NSCLC. This research suggests that TEPs' high *FLNA* expression level may be utilised to screen for high‐risk NSCLC.

## 2 Methods

**FIGURE 1:**
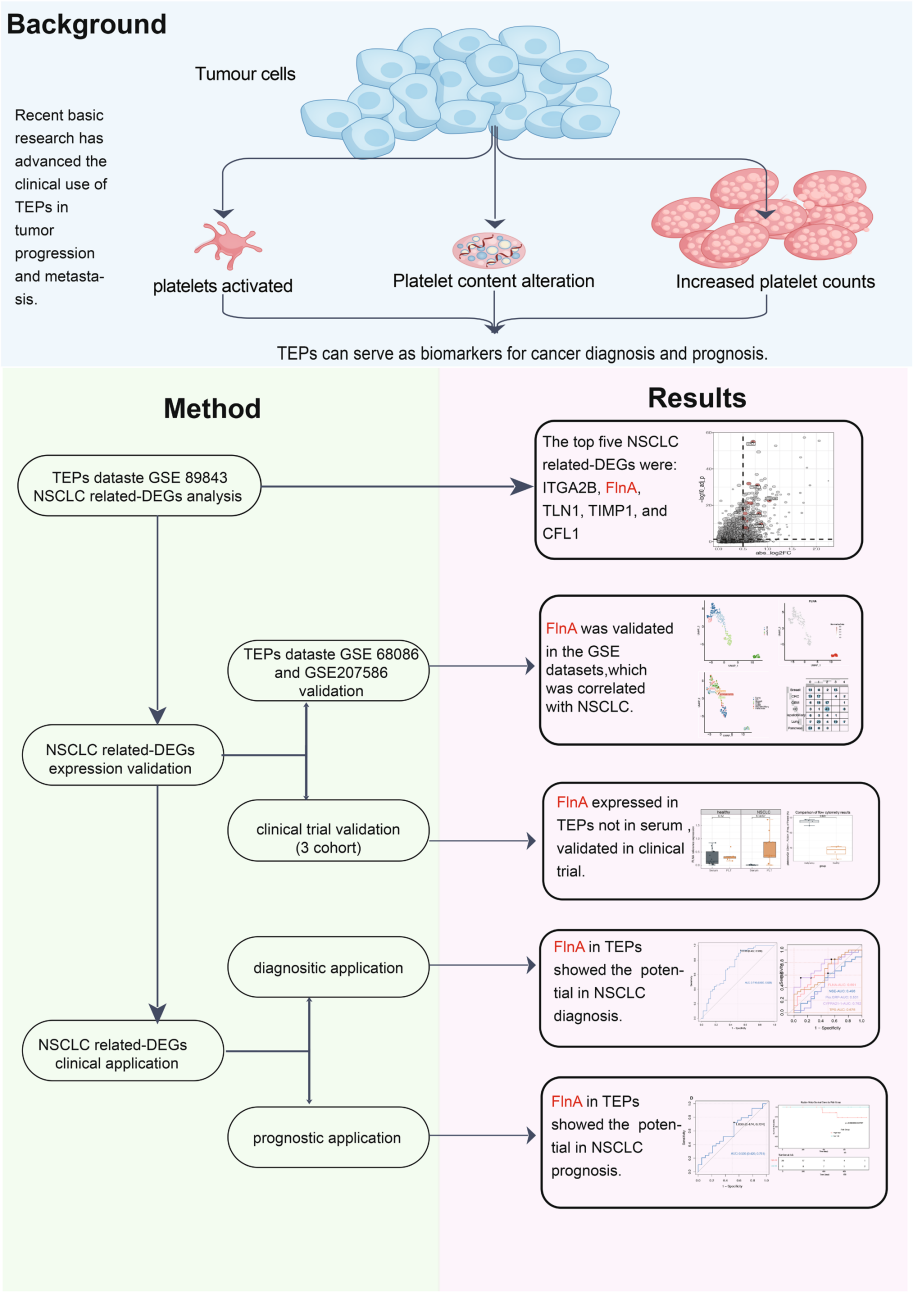
Schematic diagram of the experimental workflow, which includes the background, method, and main results. Background: Based on the previous research, TEPs could be applied to diagnose and prognosticate cancer [8]. Method: At first, the NSCLC‐specific gene was selected based on the GSE datasets; then, the expression of the specific gene was validated in datasets and a clinical trial; at last, the diagnosis and prognosis value of the specific gene was applied in datasets and a clinical trial. Results: *FLNA* was the NSCLC‐specific gene correlated with the incidence and metastasis of NSCLC, which could be used to evaluate the risk of NSCLC.

At first, the differentially expressed genes (DEGs) related to NSCLC in TEPs were screened. Then, the expression of a specific DEG was validated using datasets and clinical cohorts. Lastly, the diagnostic and prognostic efficacy of *FLNA* in NSCLC was evaluated. The workflow is shown in Figure 1. This study was approved by the medical ethical committee of Sichuan Cancer Hospital (SCCHEC‐02‐2020‐043).

### 2.1 Data Collection and Bioinformatics Analysis

We obtained the NSCLC platelet RNA profile by using the dataset of GSE89843, which was available in the public repository of the Gene Expression Omnibus (GEO) database (https://www.ncbi.nlm.nih.gov/geo/). The data set consisted of 402 platelet samples from NSCLC patients in different stages and 377 from healthy subjects. According to the different expressions of *P* value ≤ 0.05, |log2 Foldchange| ≥ 0.5 and the intra‐group mean value of the coefficient of variation (CV) ≤ 20%, the differential expressed genes were screened. Platelet sequencing data were corrected by RPKM (reads per kilobase of transcript per million reads mapped), and finally, the expression levels of differentially expressed genes we selected were compared. Besides, we used the GSE68086 dataset, which includes RNA sequencing data from 283 platelet samples across six malignant tumours, and the GSE207586 dataset, which contains RNA‐seq data from 766 platelet samples, including 399 NSCLC patients at different stages (I‐IV) and 367 healthy controls, to validate gene expression (https://www.ncbi.nlm.nih.gov/geo/). Uniform manifold approximation and projection (UMAP) was used for efficient clustering of heterogeneous samples from the sequencing data analysis. The association of differential genes with NSCLC was performed by using FindAllMarkers in the Seurat R package with the DESeq2 algorithm [15]. The transcriptome sequencing data of 1141 NSCLC samples were downloaded from TCGA (https://portal.gdc.cancer.gov/).

### 2.2 Patients and Sample Collection

This study prospectively collected platelet samples from individuals undergoing routine health examinations and patients admitted to the thoracic surgery department for the first time with diagnoses of lung nodules or lung cancer at Sichuan Cancer Hospital. Participants were categorised into the following groups based on clinical and pathological criteria:

Healthy group: Individuals were included in the healthy group if they had no detectable lung nodules or lung cancer based on LDCT screening. Additionally, all routine health examination indicators, including biochemical and haematological parameters, were required to fall within normal reference ranges.

Benign lung nodule group: Participants diagnosed with lung nodules were included in the benign lung nodule group if their admission to the thoracic surgery department was based on this diagnosis, and subsequent histopathological examination of biopsy or postoperative tissue confirmed a non‐malignant outcome.

Lung cancer group: Patients diagnosed with lung cancer and admitted to the thoracic surgery department for the first time were included in the lung cancer group. The diagnosis of lung cancer was confirmed through histopathological examination of biopsy or postoperative specimens.

The participants were excluded as follows: (1) patients who received treatments influencing platelets like blood transfusion and aspirin; (2) pregnant patients; (3) patients with infections; and (4) patients who lacked information on basic clinical characteristics.

### 2.3 Platelet Isolation and Assessment of Platelet Purity

1.5 mL of EDTA anticoagulated blood was added to 2 mL EP tubes. Platelet‐rich plasma (PRP) was separated from nucleated blood cells by centrifuging at 120 × g for 20 min (Co. Ltd. Shuke, Sichuan, China). To avoid leukocyte and erythrocyte contamination, only the upper 4/5 of the PRP was collected for further processing of platelets. The platelets were separated from PRP by centrifuging at 360 × g for 20 min. The upper strata of platelet‐poor plasma (PPP) was transferred to a new EP tube and centrifuged at 3000 g for 15 min before being frozen. To minimise platelet RNA degradation due to temporal effects, the isolation was to be completed within 2 h after blood collection. Platelet purity was determined by morphometric experiments and platelet counting by the standard of less than five white blood cells per million platelets.

### 2.4 RNA Isolation and cDNA Synthesis

Total RNA isolation used a total RNA rapid extraction kit for blood liquid samples (centrifugal column type) (Bioteke, China), following the manufacturer's instructions. The assessments of the concentration and quality of the total RNA were carried out by using a Thermo Scientific NanoDrop 2000 Spectrophotometer (Thermo Scientific, USA). Reverse transcription was carried out using a Prime Script RT reagent kit with a gDNA eraser (Takara Bio, Dalian, China), following the manufacturer's instructions.

### 2.5 Quantitative RT‐PCR (RT‐qPCR)

The primers were designed and synthesised by Tsingke Biological Technology (Beijing, China). The measurements were assessed with ΔCT normalised by GAPDH. The primers that we used are listed as follows: *GAPDH* (forward: GGAGCGAGATCCCTCCAAAAT; reverse: GGCTGTTGTCATACTTCTCATGG) and *FLNA*‐1 (forward: TCATTCGTGCCCAAGGAGAC; reverse: CTGACCAGAGACCCGAACAC). RT‐qPCR was carried out using the CFX Connect Real‐Time PCR Detection System (Bio‐Rad; Shanghai, China), in which the amplification and detection steps were combined. Reactions were performed using the TB Green Premix Ex Taq II PCR kit (TaKaRa; Dalian, China). All the assays were performed using three biological replicates. A single qPCR reaction was performed in a 20 μL volume containing 10 μL SYBR Green Master Mix, 0.8 μL of each primer, 2 μL of cDNA sample, and 6.4 μL water free of RNase and DNase. According to the manufacturer's instructions, the reactions were incubated at 95°C for 2 min 30 s, followed by 40 cycles of 95°C for 5 s and 60°C for 30 s. We calculated the amplification efficiency of the primer, which needs to exceed 95%, and analysed the melting curve of the RT‐qPCR product. The specificity of the primer is better when the melting curve shows a single peak.

### 2.6 Haematological Parameters and Serum Tumour Marker Detection Methods

Haematological parameters, including platelet count (PLT), mean platelet volume (MPV), platelet distribution width (PDW), and plateletcrit (PCT), as well as red blood cell (RBC) count, white blood cell (WBC) count, and serum platelet count, were measured using the BC‐5390 and BC‐6800 automated haematology analysers (Mindray; Shenzhen, China). The instruments underwent daily internal quality control and weekly calibration, with all results meeting acceptable criteria.

Serum tumour markers, including CEA, Pro‐GRP, NSE, and CYFRA21‐1, were detected using the Mindray CL‐8000i chemiluminescence analyser (Mindray; Shenzhen, China). The tissue polypeptide‐specific antigen (TPS) was measured using the Sorin XL chemiluminescence immunoassay analyser (DiaSorin; Italy). Both analysers underwent routine daily quality control, ensuring reliable and accurate results.

### 2.7 Statistical Data Analysis

Data analysis was performed by using the statistical programming language R (R Foundation for Statistical Computing, http://www.Rproject.org). T‐tests or nonparametric tests were used to compare the differential expression levels of *FLNA* mRNA in platelets; a *p*‐value < 0.05 was considered to be statistically significant. The diagnostic value of TEP *FLNA* mRNA was evaluated by ROC analysis.

## 3 Results

### 3.1 A Gene Linked to the Development and Progression of Lung Cancer May Be Present in Platelets Called *FLNA*

**FIGURE 2:**
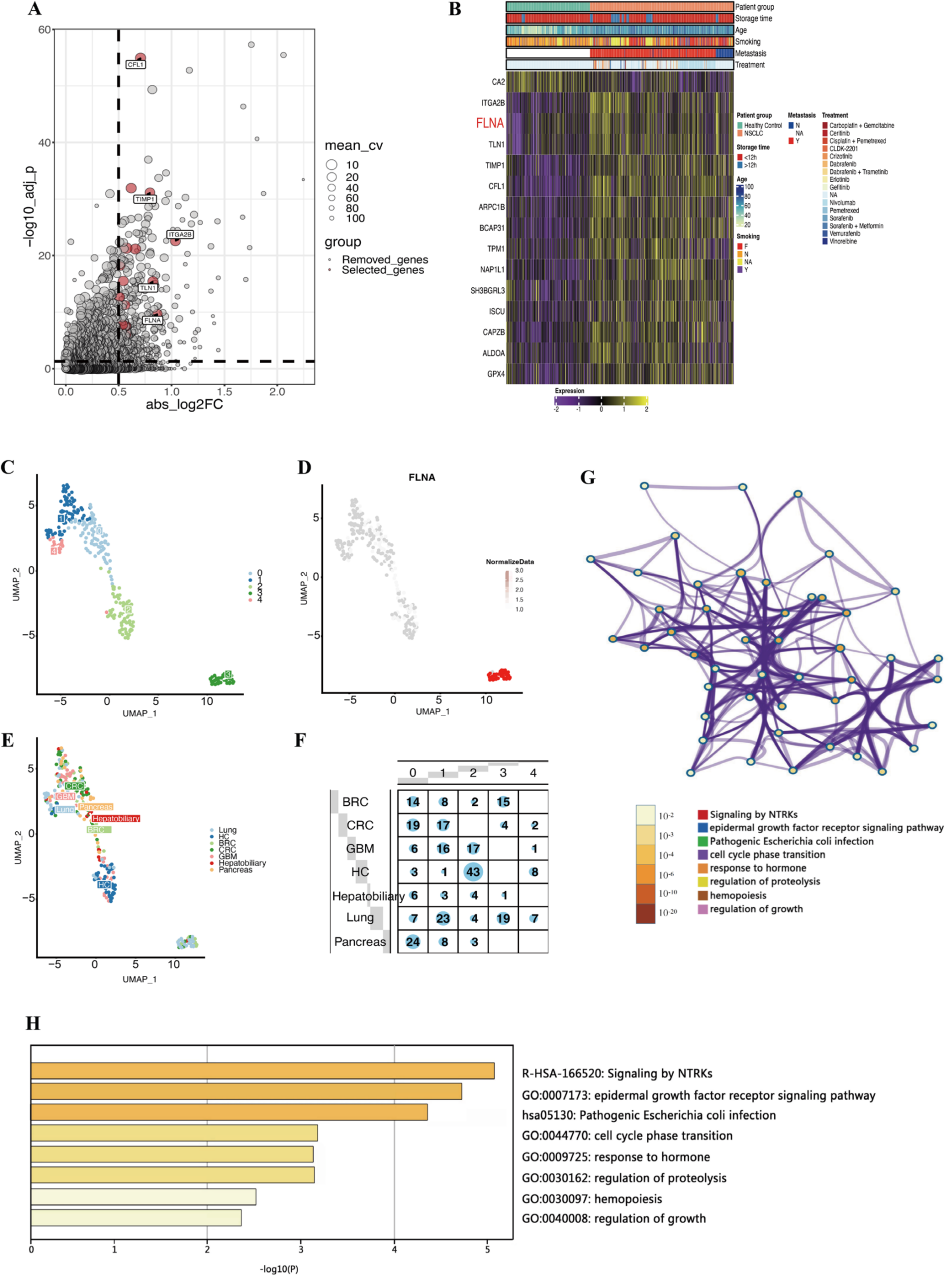
Identification of NSCLC‐specific TEP genes. Volcano plots (A) and heatmaps(B) of candidate mRNA differential genes in platelets from NSCLC patients and healthy donors based on the GSE89843. (C–F) UMAP analysis of sequencing data from 283 platelet samples and six different types of malignancies: NSCLC, CRC, pancreatic cancer (PCA), Glioblastoma multiforme (GBM), BRC and hepatobiliary cancer. (C) 283 samples are divided into five special clusters (0–4), the cluster 3 is the most special. (D) *FLNA* mRNA is mainly expressed in cluster 3. (E, F) The sample of mainly distributed individuals in cluster 3 were NSCLC and BRC individuals. (G, H) Gene functions of the top 30 highly expressed genes of the average LogFC in cluster 3 were annotated using the Metascape website. (G) Top 30 highly expressed genes enrichment network. (H) Top 30 highly expressed genes enrichment bar graph.

To search for DEGs in NSCLC platelets versus healthy donors, we analysed high‐throughput sequencing data (GSE89843) from platelet RNA samples from 402 NSCLC patients and 377 Healthy individuals. Based on the |log2FC|, the top five DEGs were printed on the volcano figure: *ITGA2B, FLNA, TLN1, TIMP1*, and *CFL1* (Figure 2A). Correlation analysis was then performed on the top 15 genes concerning treatment approaches, smoking status, metastasis, and patient subgroups. The heatmap results showed that the 15 DEGs in platelets could clearly distinguish NSCLC patients from healthy individuals (Figure 2B). In addition, GSE68086 was performed for dimensional reduction analysis in the R software package. The data were categorised into five clusters (0–4), with cluster 3 standing out as a distinct group, while the other clusters are less distinguishable and tend to group together (Figure 2C). Besides, our study results showed that TEP *FLNA* was mainly expressed in cluster 3 (Figure 2D). Then, we analysed the sample of mainly distributed individuals in the third group, and the result displayed that the majority of this group were NSCLC and breast cancer individuals (Figure 2E,F), with the highest proportion of NSCLC. Then, we performed annotated gene function for the average |log2FC| top 30 highly expressed genes in cluster 3 by using the Metascape website (https://metascape.org/gp/index.html). The results show that cluster 3 of highly expressed top 30 genes are mainly involved in the signalling by neurotrophin receptor tyrosine kinases (NTRKs), epidermal growth factor receptor (EGFR) signalling pathway, cell cycle phase transition, and growth regulation (Figure 2G,H). Therefore, according to the results, we suspected that *FLNA* expression strongly correlated with NSCLC diagnosis.

### 3.2 *FLNA* Expression Is High in TEPs of NSCLC, According to Data Gathered From Datasets

**FIGURE 3:**
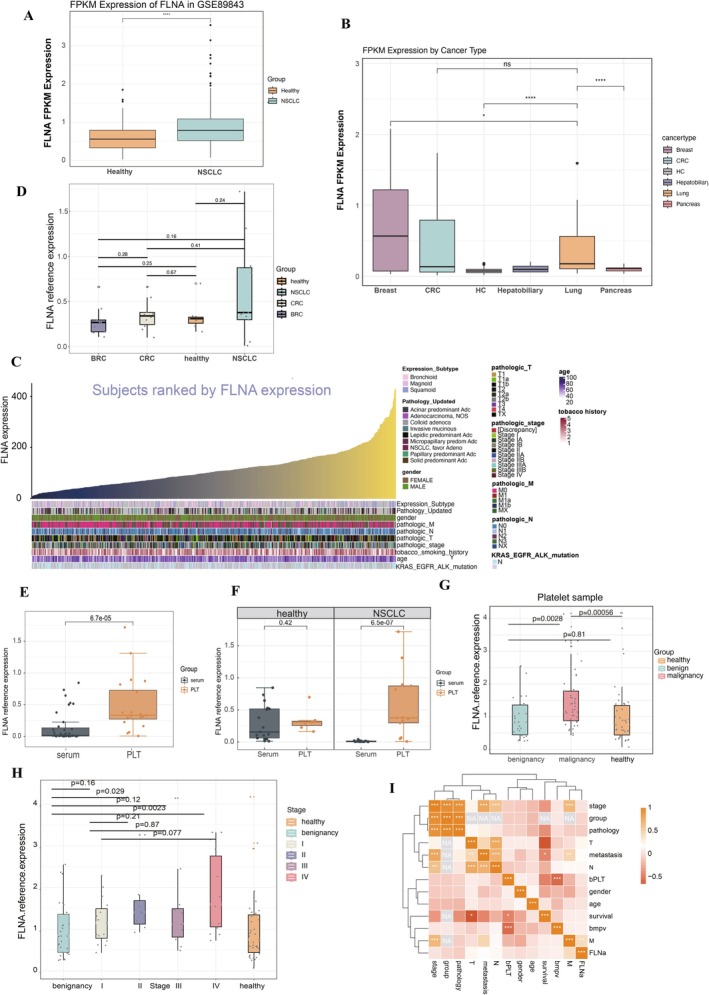
The validation of the expression levels of platelet *FLNA* mRNA. (A) The expression of *FLNA* in platelets of NSCLC and healthy donors using the FPKM to normalise the expression levels data based on GSE89843 data. (B) The FPKM expression of *FLNA* in platelets of different cancers is based on the GSE68086 data. (C) Frequency distribution histograms based on gender, age, smoking status, pathologic typing, staging, and mutation status of NSCLC patients in the TCGA database. (D) The reference expression of *FLNA* in clinical cohort 1 including 9 BRC, 10 CRC, 7 healthy, and 13 NSCLC patients. (E‐I) Relative *FLNA* mRNA expression levels in the clinical trial. (E) The comparison of relative levels of the *FLNA* mRNA in clinical cohort1 including platelets (*n* = 20) and serum (*n* = 35) from 17 healthy and 18 NSCLC participants, *p ≤ 0.05. (F) The comparison of relative levels of the *FLNA* mRNA from NSCLC (*n* = 18) between PLT (*n* = 13), and serum samples (*n* = 18), *p* < 0.05; The comparison of relative levels of the *FLNA* mRNA from healthy participants (*n* = 24) between PLT (*n* = 7), and serum samples (*n* = 17), *p* > 0.05; (G) The comparison of relative levels of the *FLNA* mRNA in platelets among NSCLC (*n* = 48) patients, healthy donors (*n* = 39) platelets, and benign patients (*n* = 29), **p ≤ 0.01. (H) The comparison of relative levels of the *FLNA* mRNA among different stages of NSCLC (*n* = 48) and benign patients (*n* = 29), including stage I (*n* = 15), stage II (*n* = 7), stage III (*n* = 12), stage IV (*n* = 14), and benign (*n* = 29). (I) The heatmap reflects the correlation analysis between relative levels of the *FLNA* mRNA with platelets features, pathology, stage, and metastasis in NSCLC (*n* = 48) patients, *p ≤ 0.05, **p ≤ 0.01, ***p ≤ 0.001.

To investigate the expression of *FLNA*, *TLN1*, *TIMP1*, and *CFL1* in NSCLC, we first analysed data from the TEP database (GSE89843), which showed elevated expression of these genes in NSCLC patients (Figure 3A and Figure S1). Validation in the pan‐cancer TEP database (GSE68086) revealed that *FLNA* may both be highly expressed in breast cancer (BRC), colorectal cancer (CRC), and lung cancer (Figure 3B). *TLN1* was more highly expressed in BRC than in lung cancer, while *TIMP1* and *CFL1* showed no significant differences between lung cancer and several other cancers, including pancreas, hepatobiliary, and CRC (Figure S1). In GSE207586, FLNA FPKM values showed a potential difference between Stage I NSCLC and non‐NSCLC (*p* = 0.076), while CFL1 and TIMP1 showed significant differences between Stage IV and non‐NSCLC (*p* = 0.00077, *p* = 0.0042). TLN1, however, showed no significant differences across stages (*p* > 0.1) (Figure S6). These results suggest that the elevated expression of these genes is not exclusive to NSCLC and may be present in TEPs from other cancers. Next, we investigated the expression of the *FLNA* gene in lung cancer tissues using the TCGA database, which revealed that the *FLNA* gene has an unclear expression level in NSCLC patients' tissues (Figure 3C). The histogram of frequency distribution results shows that patients with distant metastases (M1) typically have high expression of *FLNA*, whereas patients without distant metastases (M0) typically have low expression of *FLNA*. On the other hand, patients with NSCLC of all ages and genders showed the same distribution of *FLNA* expression (Figure 3C).

### 3.3 A Clinical Trial Verified the *FLNA* Expression in TEPs of NSCLC

To confirm NSCLC‐specific FLNA expression in TEPs, we collected platelet samples from 9 BRC, 10 CRC, 7 healthy, and 13 NSCLC patients to assess *FLNA* levels (Table 1), based on GSE68086 data indicating high *FLNA* expression in BRC and CRC. Validation revealed a trend of elevated *FLNA* expression in NSCLC relative to BRC, CRC, and healthy controls (Figure 3D).

**TABLE 1:** Clinical characteristics of pan‐cancer in cohort 1.

	Level	BRC	CRC	Healthy	NSCLC	*p*	Test
*N*		9	10	7	13		
Group	BRC	9 (100.0)	0 (0.0)	0 (0.0)	0 (0.0)	< 0.001	
	CRC	0 (0.0)	10 (100.0)	0 (0.0)	0 (0.0)		
	Healthy	0 (0.0)	0 (0.0)	7 (100.0)	0 (0.0)		
	NSCLC	0 (0.0)	0 (0.0)	0 (0.0)	13 (100.0)		
Stage	Healthy	0 (0.0)	0 (0.0)	7 (100.0)	0 (0.0)	< 0.001	
	I	1 (11.1)	0 (0.0)	0 (0.0)	0 (0.0)		
	II	5 (55.6)	3 (33.3)	0 (0.0)	1 (7.7)		
	III	1 (11.1)	4 (44.4)	0 (0.0)	6 (46.2)		
	IV	2 (22.2)	2 (22.2)	0 (0.0)	6 (46.2)		
Gender	Female	9 (100.0)	2 (20.0)	1 (14.3)	1 (7.7)	< 0.001	
	Male	0 (0.0)	8 (80.0)	6 (85.7)	12 (92.3)		
Pathology	Healthy	0 (0.0)	0 (0.0)	7 (100.0)	0 (0.0)	< 0.001	
	IBC	9 (100.0)	0 (0.0)	0 (0.0)	0 (0.0)		
	LUAD	0 (0.0)	0 (0.0)	0 (0.0)	7 (53.8)		
	LUSC	0 (0.0)	0 (0.0)	0 (0.0)	6 (46.2)		
	RA	0 (0.0)	9 (90.0)	0 (0.0)	0 (0.0)		
	SRCC	0 (0.0)	1 (10.0)	0 (0.0)	0 (0.0)		
Metastasis		0 (0.0)	1 (10.0)	0 (NaN)	0 (0.0)	NaN	
	0	4 (44.4)	3 (30.0)	0 (NaN)	2 (15.4)		
	1	5 (55.6)	6 (60.0)	0 (NaN)	10 (76.9)		
	x	0 (0.0)	0 (0.0)	0 (NaN)	1 (7.7)		
Age (median, IQR)		50.00 [35.00, 56.00]	70.50 [65.75, 77.25]	62.00 [59.00, 68.50]	61.00 [58.00, 66.00]	0.014	nonnorm
FLNA reference expression (median, IQR)		0.27 [0.16, 0.29]	0.34 [0.24, 0.38]	0.31 [0.26, 0.32]	0.38 [0.30, 0.87]	0.316	nonnorm
PLT (×10^9^/L, median, IQR)		202.00 [186.00, 213.00]	188.50 [150.50, 226.00]	178.00 [161.50, 196.50]	196.00 [163.00, 233.00]	0.782	nonnorm
MPV (fL, median, IQR)		10.70 [10.20, 11.80]	10.50 [9.30, 10.70]	10.70 [9.50, 11.85]	10.40 [9.60, 10.70]	0.631	nonnorm
PDW (median, IQR)		16.40 [16.40, 16.60]	16.35 [16.15, 16.40]	16.50 [16.05, 16.70]	16.20 [16.00, 16.30]	0.064	nonnorm
PCT (%, median, IQR)		0.22 [0.19, 0.23]	0.20 [0.16, 0.22]	0.18 [0.17, 0.22]	0.22 [0.17, 0.24]	0.38	nonnorm

To confirm platelet‐specific FLNA expression in NSCLC, we compared FLNA levels in serum and platelets to exclude free mRNA. RNA concentrations were measured in serum and platelets from 16 healthy individuals and 9 NSCLC patients, along with serum cell counts (platelets, WBC, and RBC) (Table S1). Platelet RNA concentrations were significantly higher than serum RNA, with no correlation observed between platelet count and RNA concentration or between serum RNA and cell counts (Figure S2). Platelet purity was confirmed through centrifugation and microscopic analysis (Figure S3). Detailed results are presented in the Data S1. Then, the *FLNA* expression levels were compared in the serum with the platelet samples. 17 healthy and 18 NSCLC subjects with no different distribution in age, gender, and group were included (Table 2). *FLNA* expression in platelets was significantly higher than in serum (*p* < 0.001) (Figure 3E). Interestingly, no significant difference in *FLNA* expression was observed between platelets and serum in healthy individuals. However, in NSCLC patients, *FLNA* expression in platelets was significantly higher compared to serum (*p* < 0.001) (Figure 3F). Next, we performed flow cytometry analysis comparing platelet samples from 4 healthy donors and 4 NSCLC patients (Table S2). The results demonstrated significantly elevated FLNA expression on platelets from NSCLC patients compared to healthy controls (*p* = 0.029). Representative flow cytometry plots are provided in Figure S4.

**TABLE 2:** Characteristics of NSCLC and healthy groups in serum and plasma samples from cohort 1.

	Level	Healthy	NSCLC	*p*	Test
*n*		17	18		
Group	Healthy	17 (100.0)	0 (0.0)	< 0.001	
	NSCLC	0 (0.0)	18 (100.0)		
Sample	PLT	0 (0.0)	0 (0.0)	NaN	
	Serum	17 (100.0)	18 (100.0)		
Stage	Healthy	17 (100.0)	0 (0.0)	< 0.001	
	II	0 (0.0)	1 (5.6)		
	III	0 (0.0)	6 (33.3)		
	IV	0 (0.0)	11 (61.1)		
Gender	Female	6 (35.3)	4 (22.2)	0.63	
	Male	11 (64.7)	14 (77.8)		
Pathology	Healthy	17 (100.0)	0 (0.0)	< 0.001	
	LUAD	0 (0.0)	11 (61.1)		
	LUSC	0 (0.0)	7 (38.9)		
Metastasis	0	0 (NaN)	2 (11.1)	NaN	
	1	0 (NaN)	15 (83.3)		
	x	0 (NaN)	1 (5.6)		
Age (median, IQR)		58.00 [38.00, 62.00]	60.00 [58.00, 66.75]	0.123	nonnorm
FLNA reference expression (median, IQR)		0.16 [0.06, 0.52]	0.01 [0.00, 0.02]	< 0.001	nonnorm
PLT(×10^9^/L, median, IQR)		218.00 [179.00, 238.00]	195.50 [167.50, 230.50]	0.4	nonnorm
MPV(fL, median, IQR)		10.70 [9.40, 11.20]	10.40 [9.62, 11.70]	1	nonnorm
PDW (median, IQR)		16.40 [16.10, 16.50]	16.20 [16.10, 16.30]	0.281	nonnorm
PCT (%, median, IQR)		0.22 [0.19, 0.24]	0.22 [0.18, 0.25]	0.644	nonnorm

Above all, the specific *FLNA* expression on platelets in NSCLC patients was validated. Thus, more samples were collected for further research. According to the exclusion and inclusion criteria, 48 NSCLC patients, 29 benign patients, and 39 healthy subjects were included in the main study to verify whether TEP *FLNA* mRNA could be a biomarker for NSCLC diagnosis (Table 3). The relative expression of *FLNA* was significantly higher in the malignant group than in both other groups, while there was no significance between the other two groups. It was discovered that NSCLC patients with stage IV had considerably higher levels of *FLNA* expression in their platelets (*p* < 0.01, Figure 3H). The *FLNA* was expressed similarly in LUAD and LUSC groups, although they deviated from the benign group with significance (*p* < 0.05, Figure S5F). Clinical correlation analysis also revealed a positive relationship between *FLNA* expression and NSCLC metastasis (*p* = 0.01, Figure 3I).

**TABLE 3:** Characteristics of healthy, benign, and NSCLC groups in cohort 2.

	Level	Benignancy	Malignancy	Normal	*p*	Test
*n*		29	48	39		
Stage	Benignancy	29 (100.0)	0 (0.0)	0 (0.0)	< 0.001	
	I	0 (0.0)	15 (31.2)	0 (0.0)		
	II	0 (0.0)	7 (14.6)	0 (0.0)		
	III	0 (0.0)	12 (25.0)	0 (0.0)		
	IV	0 (0.0)	14 (29.2)	0 (0.0)		
	Normal	0 (0.0)	0 (0.0)	39 (100.0)		
Gender	Female	17 (58.6)	16 (33.3)	25 (64.1)	0.01	
	Male	12 (41.4)	32 (66.7)	14 (35.9)		
Pathology	Benignancy	29 (100.0)	0 (0.0)	0 (0.0)	< 0.001	
	LUAC	0 (0.0)	36 (75.0)	0 (0.0)		
	LUSC	0 (0.0)	12 (25.0)	0 (0.0)		
	Normal	0 (0.0)	0 (0.0)	39 (100.0)		
Metastasis	0	0 (NaN)	19 (39.6)	0 (NaN)	NaN	
	1	0 (NaN)	29 (60.4)	0 (NaN)		
Age (median, IQR)		56.00 [51.00, 64.00]	62.50 [57.75, 68.00]	53.00 [49.00, 58.00]	< 0.001	nonnorm
PLT(×10^9^/L, median, IQR)		190.00 [143.00, 217.00]	212.00 [175.25, 230.75]	149.00 [10.65, 194.50]	< 0.001	nonnorm
MPV(fL, median, IQR)		10.40 [9.60, 11.60]	10.50 [9.50, 11.40]	12.70 [10.60, 15.85]	< 0.001	nonnorm
PDW (median, IQR)		16.30 [16.00, 16.50]	16.30 [16.00, 16.50]	16.10 [0.24, 16.30]	0.008	nonnorm
PCT (%, median, IQR)		0.20 [0.17, 0.24]	0.21 [0.19, 0.25]	0.15 [0.10, 0.17]	< 0.001	nonnorm
NSE (ng/ml, median, IQR)		12.95 [9.05, 14.35]	12.03 [9.75, 14.34]	5.80 [5.80, 5.80]	0.223	nonnorm
Pro‐GRP (pg/ml, median, IQR)		44.31 [33.97, 56.43]	45.47 [39.02, 52.11]	51.04 [51.04, 51.04]	0.843	nonnorm
CEA (ng/ml, median, IQR)		1.38 [1.15, 1.77]	2.53 [1.51, 5.52]	NA [NA, NA]	0.077	nonnorm
CYFRA21‐1 (ng/ml, median, IQR)		1.92 [1.52, 2.63]	3.31 [2.42, 6.64]	3.14 [3.14, 3.14]	0.002	nonnorm
TPS (U/L, median, IQR)		63.31 [42.29, 102.87]	92.85 [71.56, 145.12]	91.06 [91.06, 91.06]	0.062	nonnorm

### 3.4 Elevated Expression of *FLNA* in TEPs Can Be Utilised to Distinguish NSCLC From Healthy Individuals as Well as NSCLC From Benign Individuals

**FIGURE 4:**
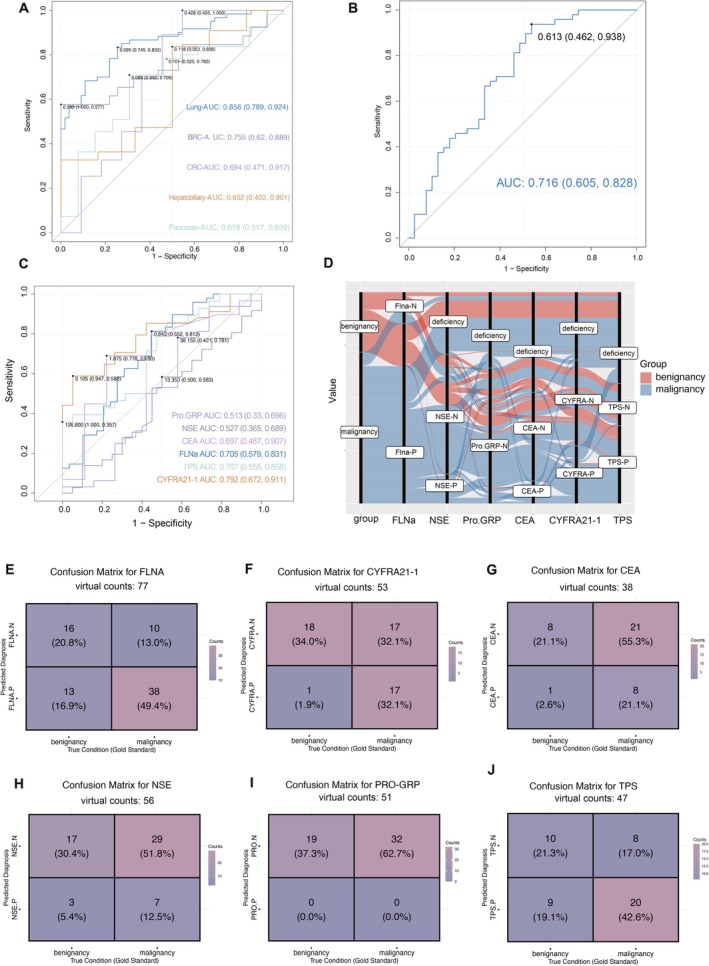
The potential of platelet *FLNA* mRNA in assessing the incidence of NSCLC. (A) ROC analysis of the platelet *FLNA* to distinguish between NSCLC, CRC, pancreatic cancer, BRC, and hepatobiliary cancer patients from healthy donors, respectively, based on GSE68086 data. (B) ROC analysis of *FLNA* mRNA in platelets to distinguish between NSCLC patients and healthy donors based on the clinical cohort2. (C) ROC analysis of platelet *FLNA* mRNA, NSE, Pro‐GRP, CYFRA21‐1, and TPS to distinguish between NSCLC and benign patients based on the clinical cohort2, respectively. (D‐J) Participants, including benign and malignant cases, were grouped into FLNA‐positive and FLNA‐negative based on a cutoff of 0.8479772. NSE, Pro‐GRP, CEA, CYFRA21‐1, and TPS were classified as positive or negative using their reference upper limits (> 16.3 ng/mL, > 77.8 pg/mL, > 5 ng/mL, > 3.3 ng/mL, > 75 U/L, respectively). (D) An alluvial plot visualises patient groupings, with missing data labelled as “deficient.” (E–J) Four‐compartment tables display FLNA expression (E), CYFRA21‐1 (F), CEA (G), NSE (H), Pro‐GRP (I), and TPS (J) classification alongside actual patient groupings.

Analysis of the GSE68086 dataset revealed that *FLNA* demonstrated superior diagnostic performance in distinguishing lung cancer from healthy controls, with an area under roc curve (AUC) of 0.856 (95% CI, 0.789, 0.924), compared to its discriminative capacity for BRC and CRC (Figure 4A). Specifically, there was a possible difference of *FLNA* expression between Stage I NSCLC and non‐NSCLC in GSE207586 (*p* = 0.076), suggesting its potential as an early‐stage biomarker. In contrast, *TLN1*, *TIMP1*, and *CFL1* exhibited better diagnostic efficacy (Figure S1) for BRC and CRC than for lung cancer in ROC analysis. In contrast, although CFL1, TIMP1, and TLN1 showed significant differences between NSCLC and healthy controls, no significant differences were found across different NSCLC stages. CFL1 and TIMP1 did show significant differences between Stage IV and non‐NSCLC (*p* = 0.00077, *p* = 0.0042), while TLN1 showed no stage‐dependent differences (*p* > 0.1) (Figure S6). These findings suggest that CFL1, TIMP1, and TLN1 have limited value in distinguishing early‐stage NSCLC, while FLNA is more effective for this purpose. Given that CFL1, TIMP1, and TLN1 have been investigated for their diagnostic potential in other cancer types, we did not pursue further exploration of their diagnostic value in NSCLC in the current study [16, 17, 18].

When *FLNA* expression was utilised to differentiate between NSCLC and healthy populations in our collected clinical cohort and ROC curves were plotted, the AUC may reach 0.716 (95% CI, 0.605–0.828) (Figure 4B). As the probability of developing NSCLC with age over 40 years increased, we corrected the selected subjects for sex and age factors by using a logistic regression model to reduce selective bias. The results also showed that the TEPs *FLNA* mRNA was an independent diagnostic factor in differentiating NSCLC patients (*p* < 0.05) (Table 4). Also, we corrected the selected NSCLC and benign subjects for sex and age factors by using a logistic regression model to reduce selective bias. The results displayed that the *FLNA* mRNA in platelet samples was an independent diagnostic factor in differentiating NSCLC patients and benign pulmonary nodules (*p* < 0.05) (Table 5). An increase in *FLNA* had a greater effect on the probability of an incidence of lung cancer than age.

**TABLE 4:** An overview of logistic regression showed the variables used to separate NSCLC patients from healthy individuals.

	Estimate	Std.error	*Z* value	*p*
(Intercept)	−12.70729	2.92747	4.341	1.42E‐05
Age	0.16519	0.04203	−3.930	8.49E‐05
Gender	1.39831	0.56894	−2.458	0.01398
*FLNA*	0.99731	0.35085	−2.843	0.00448

**TABLE 5:** An overview of logistic regression showed the variables used to separate NSCLC patients from benign patients.

	Estimate	Std. error	*Z* value	*p*
(Intercept)	−6.73056	2.19788	−3.062	2.20E‐03
Age	0.07405	0.03097	2.391	1.68E‐02
Gender	0.97838	0.53892	1.815	0.06946
*FLNA*	1.07929	0.40969	2.634	0.00843

### 3.5 *FLNA* in TEPs Exceeded the Common Serum Tumour Markers in Distinguishing NSCLC From Benign Individuals

We also investigated the role of *FLNA* in distinguishing between benignancy and NSCLC patients and compared it with 5 common tumour markers. Among the Cohort 2 samples, due to data availability, FLNA was measured in 77 patients, NSE in 56 patients, Pro‐GRP in 51 patients, CEA in 38 patients, CYFRA21‐1 in 53 patients, and TPS in 47 patients. This pragmatic approach was applied to ensure meaningful statistical analysis, while acknowledging the limitation of partial data availability. By comparing the levels of common serum tumour markers in different stages of NSCLC, CYFRA21‐1 and TPS showed ascending trends along with the ascending stages (Figure S5). As shown in Figure 4C, the pulmonary benign and malignant differentiation of the AUC for *FLNA* in platelet samples was 0.705 (95% CI: 0.579–0.831), while the CYFRA 21‐1 reached a higher AUC at 0.792 (95% CI: 0.672–0.911). To maximise the sensitivity and specificity, the best cut‐off value at 0.8479772 with a sensitivity of 0.813 and specificity of 0.552 was chosen based on the ROC curve. Benign and malignant patients were divided into *FLNA*‐positive and *FLNA*‐negative groups based on relative *FLNA* expression according to the cut‐off value of 0.8479772. NSE, Pro‐GRP, CEA, CYFRA21‐1, and TPS were classified as positive or negative based on their reference upper limits (> 16.3 ng/mL, > 77.8 pg/mL, > 5 ng/mL, > 3.3 ng/mL, > 75 U/L, respectively). An alluvial plot was generated using the correct patient groupings, with missing data labelled as “deficiency” (Figure 4D). According to the alluvial plot, we discovered that *FLNA* is relatively more accurate in determining malignancy, but some benign nodules have been misclassified as malignant. CYFRA21‐1, on the other hand, has even fewer misclassifications of malignancy but can still be malignant in close to half of the benign individuals it determines.

Comparative analysis of diagnostic performance between *FLNA* and conventional serum tumour markers in distinguishing NSCLC from benign pulmonary nodules revealed distinct patterns (Figure 4E–J). *FLNA* demonstrated superior sensitivity (TPR = 0.792) compared to conventional markers, particularly outperforming CYFRA21‐1 (0.500), CEA (0.276), NSE (0.194), and Pro‐GRP (0.000). While CYFRA21‐1 showed the highest specificity (TNR = 0.947) and positive predictive value (PPV = 0.944) among all markers, its sensitivity was substantially lower than *FLNA* (Table S3). Notably, *FLNA* maintained balanced diagnostic performance across all parameters, with moderate specificity (TNR = 0.552) and predictive values (PPV = 0.745, NPV = 0.615) (Table S3). These findings suggest that *FLNA* may serve as a more sensitive biomarker for initial screening, whereas conventional markers, particularly CYFRA21‐1, could be valuable for confirmatory diagnosis due to their high specificity. A simple logistic regression analysis developed a combined predictive value using FLNA and CYFRA21‐1 (Table S4). Our results showed significant differences between NSCLC and benign lung nodules as well as across different NSCLC stages (Figure S7). The combined predictive value demonstrated promising diagnostic performance, with an AUC of 0.839 for distinguishing NSCLC from benign lung nodules and 0.709 for distinguishing metastatic from non‐metastatic NSCLC (Figure S7).

### 3.6 TEPs With Elevated *FLNA* Expression Could Be Used to Assess Metastasis and Survival Prognosis in NSCLC Patients

**FIGURE 5:**
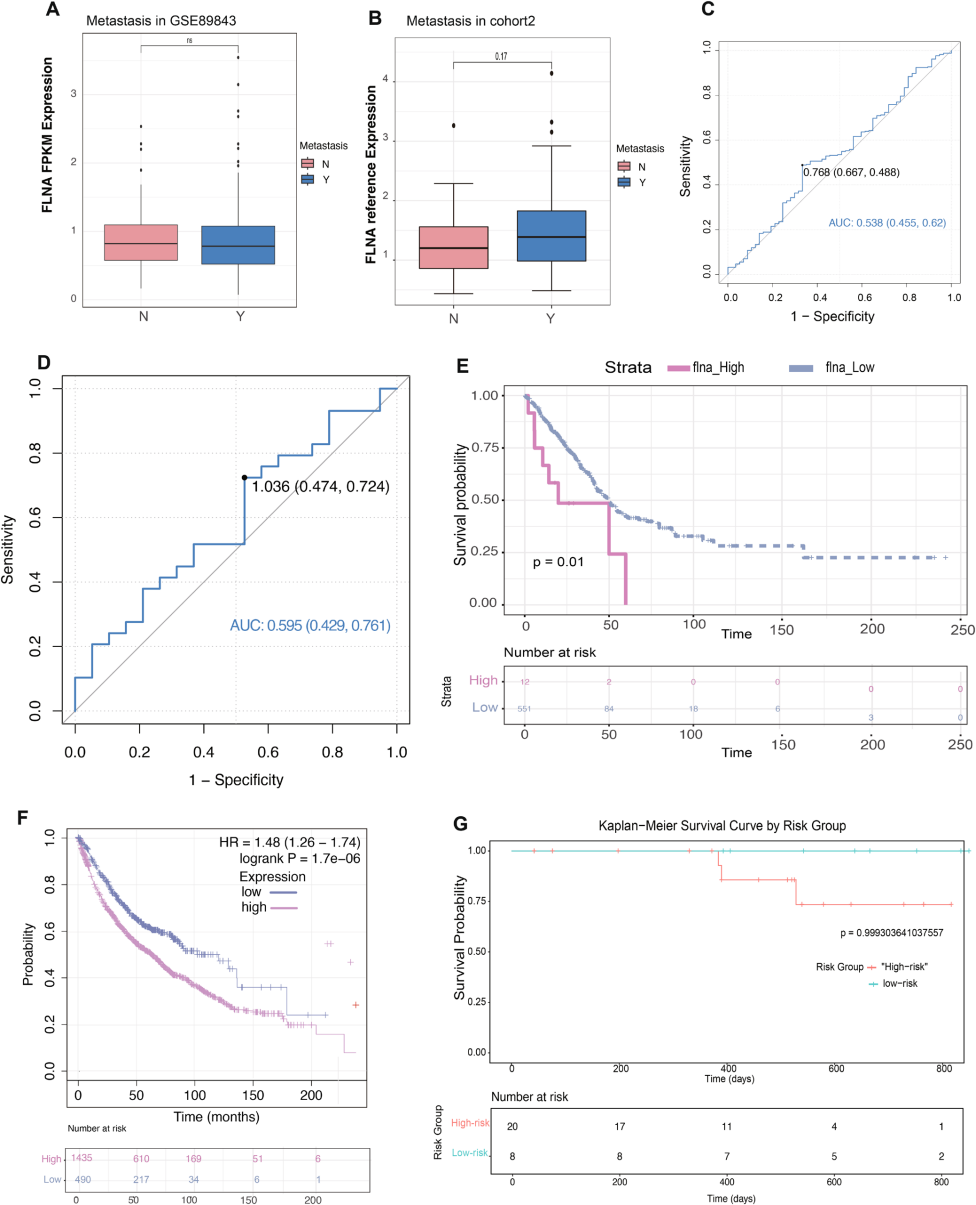
The potential of platelet *FLNA* mRNA in assessing the metastasis and survival prognosis of NSCLC. (A) A boxplot compares *FLNA* expression in metastatic and non‐metastatic NSCLC patients using GSE89843 datasets. (B) A boxplot compares *FLNA* expression in metastatic and non‐metastatic NSCLC patients based on our clinical cohort 2 results. (C) ROC analysis of the platelet *FLNA* to distinguish between metastatic and non‐metastatic NSCLC patients based on GSE89843 datasets. (D) ROC analysis of *FLNA* mRNA in platelets to differentiate between metastatic and non‐metastatic NSCLC patients based on the clinical cohort 2. (E) The K‐M plot reflects the survival prognosis of NSCLC patients based on the *FLNA* expression in TCGA datasets. (F) The K‐M plot reflects the survival prognosis of NSCLC patients based on the *FLNA* expression using the Kaplan–Meier Plotter database. (G) The K‐M plot reflects the survival prognosis of NSCLC patients based on the *FLNA* expression using the results of our clinical trial.

Our investigation into the prognostic value of *FLNA* in NSCLC revealed limited efficacy in predicting metastatic potential. Analysis of both the GSE89843 dataset and our validation cohort (cohort 2) demonstrated a non‐significant elevation of *FLNA* expression in metastatic NSCLC patients compared to non‐metastatic cases (Figure 5A,B). While analysis of the GSE89843 dataset showed limited discriminative capacity (AUC = 0.538, 95% CI:0.455–0.62) (Figure 5C), cohort 2 demonstrated modest improvement in diagnostic performance (AUC = 0.595, 95% CI: 0.429–0.761) (Figure 5D). Also, a logistic regression model displayed that the *FLNA* mRNA in platelet samples was not an independent diagnostic factor in differentiating metastasis patients and non‐metastasis patients (*p* > 0.05) (Table 6). This differential performance suggests potential context‐dependent utility of *FLNA* that may be influenced by cohort characteristics or disease heterogeneity. Although falling short of strong predictive value (AUC > 0.7), the cohort 2 results indicate that *FLNA* might contribute the incremental value to composite biomarkers for metastasis risk stratification. Further investigation is warranted to clarify its role in specific molecular subtypes or therapeutic response prediction.

**TABLE 6:** An overview of logistic regression showed the variables used to separate metastasis patients from non‐metastasis patients.

	Estimate	Std. error	*Z* value	*p*
(Intercept)	−0.83665	2.84656	−0.294	0.769
Age	0.02245	0.04080	0.550	0.582
Gender	−0.56041	0.66596	−0.841	0.4
*FLNA*	0.5599	0.41871	1.337	0.181

TCGA analysis results demonstrated a negative correlation of *FLNA* expression with the survival prognosis of patients (Figure 5E). Using the Kaplan–Meier Plotter database to investigate the survival predictive value of *FLNA* in lung cancer, it was discovered that patients with higher expression had a poorer prognosis (Figure 5F). The optimal cut‐off value (1.0431263) was determined by analysing the ROC curve, which distinguished between metastasis and non‐metastasis based on the relative expression of *FLNA*. Patients with NSCLC who had *FLNA* expression higher than the optimal cut‐off value were classified as high‐risk, while those with expression less than or equal to the optimal cut‐off value were classified as low‐risk. The K‐m curves were displayed alongside patient survival, showing a trend toward a lower 2‐year survival rate for the high‐risk group. However, due to the small sample size, this result did not reach statistical significance (Figure 5G).

## 4 Discussion

Lung cancer is one of the most common cancers worldwide, with an increasing incidence, and has long been a disease characterised by advanced diagnosis [19, 20]. As reported by the national lung cancer screening guidelines in China, LDCT is recommended as the primary means of screening for NSCLC [21]. On the other hand, cytologic puncture, surgery, or LDCT follow‐up will be used to confirm the diagnosis for patients whose lung nodules are not apparent by LDCT. The guidelines also suggest that various indicators can review the high‐risk category to prevent unneeded tests and treatments, as this will likely cause radiation harm, physical damage, and psychological and financial pressure on the patients.

Various studies have shown that liquid biopsy is considered an important tool for early cancer detection and noninvasive tumour analysis and monitoring of cancer course due to its advantages of continuous sampling, automated completion, and reproducible results [22, 23]. Platelets are highly considered an emerging biological source of liquid biopsy, which has several advantages as biomarkers for cancer diagnosis, including their abundance, easy isolation, relatively higher RNA quality, and their ability to respond to external signals to process RNA [24]. As reported, patients' circulating cancer cells absorb platelets and transfer lipids, RNA, and proteins to the cancer cells, promoting the stemness and proliferative potential of the cancer cells [25]. On the other hand, many observations suggested that platelets can educate cancer cells and regulate and absorb tumour RNA signals, leading to changes in the platelet transcriptomic profile that can reflect the pathological progression [26]. In 2010, the platelets mRNA profiles between healthy donors and 5 untreated metastatic lung cancer patients have been analysed. The results revealed significant alterations in 200 genes, including 197 genes with low expression [27]. Subsequently, Nilsson found the cancer‐associated RNA biomarkers, including EGFRvIII and PCA3, from isolated platelets from glioma and prostate cancer patients and demonstrated that tumour cells can transfer RNA to platelets [24]. That being said, TEP mRNA has emerged as a promising novel biomarker for cancer diagnosis, prognosis, and prediction. It can be particularly useful for identifying individuals who are at high risk of developing non‐small cell lung cancer.

Since TEPs are tumour‐specific and generated via education of tumour cells, the proteins, lipids, and nucleic acids within them serve as indicators that may react differently to distinct tumour types. The TEP sequencing‐based databases GSE89843 and GSE68086 were our first step while seeking genes that are particular to NSCLC. In our research, 15 DEGs in platelets were selected between NSCLC patients and healthy individuals, and the top 5 DEGs included *ITGA2B, FLNA, TLN1, TIMP1, and CFL1*. The high expression of TEP *ITGA2B* mRNA in NSCLC has been verified [28]. The preliminary validation of the same study has shown that *TIMP1* is expressed higher in the NSCLC patients when compared to the benign nodule patients, but little difference in expression was observed compared to healthy individuals, while *TLN1* provided poor discriminatory performance for NSCLC and healthy individuals [17]. Previous studies have highlighted the roles of *TLN1*, *TIMP1*, and *CFL1* from platelets in various cancers and platelet‐associated disorders beyond NSCLC. For instance, *TLN1* is critical for integrin activation mediated by its interaction with the β3 cytoplasmic tail following *P*‐selectin binding and is essential for endothelial proliferation and postnatal angiogenesis [29]. Additionally, *TIMP1* mRNA in tumour‐educated platelets has been validated as a diagnostic biomarker for colorectal cancer, while independent studies have established reliable reference genes for platelet transcripts in myocardial infarction patients and identified potential plasma biomarkers for acute myocardial infarction via proteomics [17]. CFL1 is stably highly expressed in platelets from both healthy individuals and patients with myocardial infarction, making it unsuitable as a diagnostic biomarker for NSCLC either [30]. The validated results in GSE207586 suggest that CFL1 and TIMP1 may be more relevant to later‐stage NSCLC, while FLNA shows promise as a potential marker for early‐stage diagnosis. These findings indicate that FLNA, unlike CFL1, TIMP1, and TLN1, might have greater utility as an early‐stage diagnostic biomarker for NSCLC. This supports our decision not to explore the diagnostic and prognostic value of TLN1, CFL1, and TIMP1 further in this study, as their expression patterns suggest limited specificity for early‐stage NSCLC.

The role of TEP *FLNA* mRNA in NSCLC was not verified in other studies, and our analysis showed that it might be a key differential gene in NSCLC. In summary, we selected *FLNA* as the most appropriate differentially expressed gene for inclusion in subsequent validation. Filamin A *(FLNA)* is a well‐known actin cross‐linking protein involving a variety of cellular functions [31]. It is a scaffold for more than 90 binding partners, including channels, receptors, and intracellular signalling molecules. Therefore, mutations in the human *FLNA* genes are associated with a wide range of diseases, including many kinds of cancer [32]. For example, *FLNA* has been shown to be overexpressed in prostate cancer (PC) [33] and altered in CRC tissues, which was significantly associated with poor lymph node metastasis, clinical stage, histological grade, and overall survival [32]. Interestingly, *FLNA* was originally found to be a pro‐oncoprotein but played a dual role in cancer [34]. For example, a dual role of *FLNA* in the metastasis of breast cancer has been proven. On the one hand, highly expressed *FLNA* regulates the breakdown of focal adhesion and inhibits the migration and invasion of breast cancer cells [35, 36]. On the other hand, the absence of *FLNA* in breast cancer cells significantly reduces their migration and invasion through their interaction with Cyclin D [37]. More importantly, plasma *FLNA* was a specific and sensitive marker in patients with metastatic breast cancer [38]. At present, the research on *FLNA* and cancer is mainly based on plasma and tissue. There has been no special research on the relationship between *FLNA* expression in platelets and cancer.

Again, after evaluating the profile data, we discovered that *FLNA* was elevated only in the TEPs of NSCLC patients, whereas *FLNA* expression in the TEPs of breast cancer patients was identical to that of the healthy population. This implies that *FLNA* might be a particular marker for TEPs in patients with NSCLC. After being validated in the clinical cohort, it was also suggested that the expression of *FLNA* mRNA was significantly elevated in platelets from NSCLC patients. Since plasma may contain platelets and leukocytes, the *FLNA* detected in plasma could originate from these cells rather than representing free circulating mRNA. Therefore, to eliminate the potential influence of free mRNA, we first measured the white blood cell and platelet counts in serum before examining *FLNA* expression in serum. Our results showed that there were almost no platelets or leukocytes in the serum. In healthy individuals, *FLNA* expression was similar in both serum and platelets, whereas in NSCLC patients, *FLNA* expression in platelets was higher than that in serum. Combined with our flow cytometry findings, *FLNA* expression was significantly higher in the platelets of NSCLC patients compared to healthy individuals. This suggests that *FLNA* may serve as a specific marker for TEPs in NSCLC and could potentially be used to predict the risk of NSCLC development. Importantly, we also found that the platelet *FLNA* mRNA expression in platelets of benign and malignant lung nodules was still significantly different. Although *FLNA* expression in platelets has not been explicitly shown to vary between sexes, we chose to correct for sex in the statistical analysis as a precautionary measure. Previous studies have demonstrated that various platelet‐related parameters, as well as molecular expressions, can exhibit sex‐based differences [39, 40]. Given that biological sex can influence the expression of certain genes and proteins, it was important to account for this potential source of variability in our data. By correcting for sex, we aimed to ensure that any observed differences in *FLNA* expression were not confounded by sex‐related factors.

Besides, our experimental results showed that TEP *FLNA* mRNA can not only deliberately differentiate NSCLC patients from healthy donors but also deliberately differentiate benign and malignant lung nodules. The ROC results showed a good diagnostic efficiency with AUC over 0.7. Of course, there were some limitations to our study, such as the small sample size. It may lead to the diagnostic model results not being perfect as imagined and may require more sequencing and RT‐qPCR validation in larger groups to determine the final diagnostic efficacy in the future. However, we cannot deny that *FLNA* mRNA in TEPs could be provided as a potential biomarker for assessing the risk of NSCLC, which also exceeded the common serum biomarkers. For the serum biomarkers Pro‐GRP, NSE, CEA, TPS, and CYFRA 21‐1, the thresholds were set at the upper limits of their respective clinical biological reference intervals. These reference intervals are typically established by the manufacturers of diagnostic reagents and clinical laboratories, using data from large sample sizes of healthy individuals. They represent the range in which most healthy individuals fall. Values exceeding the upper limit of these intervals are generally interpreted as indicative of a potential risk for cancer, as they may suggest abnormal levels of these biomarkers. Similarly, for the *FLNA* mRNA‐based ROC curve, the cutoff value was selected to represent the threshold above which the risk for cancer increases. By using this approach, we compared the performance of serum tumour markers with *FLNA* mRNA expression levels, allowing for a direct comparison of their diagnostic potential. However, we recognise the potential value of combining FLNA mRNA expression with CYFRA 21‐1, even through a simple logistic regression. The combination of these two markers not only accurately distinguishes NSCLC from benign nodules but also effectively differentiates between the four stages of NSCLC and shows strong efficiency in distinguishing metastatic from non‐metastatic cases.

Nevertheless, the construction of a robust model requires a rigorous modelling and validation cohort. Due to the small sample size, the results of this simple logistic regression model may not be fully reliable. Future studies should aim to expand the sample size and use both a modelling and validation cohort to explore more effective modelling approaches, enhancing the sensitivity, specificity, and accurate differentiation of FLNA for NSCLC patients across the four stages.

Correlation research led us to the conclusion that metastasis and *FLNA* expression in TEPs might be interrelated. Thus, we investigated further *FLNA* expression in databases and clinical cohorts containing both metastatic and non‐metastatic NSCLC patients. In this study, we observed that the AUC for discriminating metastasis from non‐metastasis (Figure 5D) was 0.595, with a 95% CI ranging from 0.429 to 0.761. The fact that the confidence interval crossed the threshold of 0.5 suggests that the predictive performance of this marker may not be robust and could reflect variability within the sample population rather than a true diagnostic ability. This limitation may arise from the relatively small sample size or heterogeneity among patients. Despite this, the observed trend provides preliminary evidence for potential clinical utility, which warrants further investigation. Future studies with larger cohorts and more rigorous validation are needed to confirm the diagnostic value of this marker and to refine its application in clinical settings.

When platelets come into direct contact with cancer cells, they become activated and form microaggregates around the surface of the tumour, shielding the tumour cells from immune identification [41]. Remarkably, several recent investigations have demonstrated that cancer cells can swallow whole platelets in a dynamin‐dependent phagocytosis or membrane fusion‐dependent way when they come into contact with platelets [25, 42]. According to these results, tumour cells can perform a “platelet mimicry,” which involves absorbing and presenting or using proteins, nucleic acids, and lipids produced from platelets to evade the immune system and increase their potential for growth and metastasis. The study's findings suggest that *FLNA* is a crucial TEP molecule in NSCLC metastasis, and it would be beneficial to investigate the molecular mechanism of this molecule's potential role in metastasis in further research.

As metastasis in NSCLC is frequently positively correlated with a poor prognosis for survival, and since *FLNA* in TEPs is linked to metastasis, we postulated that *FLNA* in TEPs may also be utilised to predict survival in NSCLC patients. We subsequently analysed the relationship between NSCLC patient survival prognosis and *FLNA* mRNA expression levels through the Kaplan–Meier Plotter website (http://kmplot.com/analysis/) and TCGA datasets. The results showed that high *FLNA* expression can influence the patient's prognosis. In this study, we observed a trend toward a lower 2‐year survival rate in the high‐risk group, suggesting the potential prognostic value. However, it is important to note that due to the small sample size, this finding did not reach statistical significance. Due to the limited sample size overall and the fact that the majority of the patients included in this trial had not yet reached the 3‐year follow‐up, we cannot verify whether TEP *FLNA* mRNA expression is associated with NSCLC patient survival prognosis. We will follow up with a close visit to verify whether *FLNA* in TEPs is related to NSCLC in the future.

## 5 Conclusions

This study compares the expression of the platelet *FLNA* in NSCLC patients, patients with benign lung nodules, and the healthy population. It shows that high expression of *FLNA* is linked to the development of NSCLC and can distinguish NSCLC from benign lung nodules or healthy individuals with a diagnostic efficiency that is better than several serum tumour markers currently in use in clinical practice. It suggests that detecting the expression of platelet *FLNA* can help to determine the high‐risk population of NSCLC and reduce the positive misdiagnosis rate of LDCT. In the meantime, we also discovered that the platelet *FLNA* was linked to NSCLC metastasis. This finding may not only point to a correlation with the incidence of NSCLC but also a potential platelet participation mechanism in NSCLC metastasis, which naturally has to be investigated further through our basic study.

## Author Contributions

**Ruiling Zu:** conceptualization (equal), data curation (equal), formal analysis (equal), funding acquisition (lead), investigation (equal), methodology (equal), project administration (equal), supervision (equal), writing – original draft (equal). **Hanxiao Ren:** methodology (equal). **Xing Yin:** investigation (equal). **Lubei Rao:** writing – review and editing (equal). **Xingmei Zhang:** formal analysis (equal), methodology (equal). **Pingyao Xu:** methodology (equal). **Dongsheng Wang:** supervision (equal), visualization (equal), writing – review and editing (equal). **Yuping Li:** writing – review and editing (equal). **Huaichao Luo:** conceptualization (equal), funding acquisition (equal), writing – review and editing (equal).

## Ethics Statement

*Approval of the research protocol by an Institutional Review Board*: This research was approved by the medical ethical committee of Sichuan Cancer Hospital (SCCHEC‐02‐2020‐043).

*Informed Consent*: N/A.

*Registry and the Registration No. of the study/trial*: N/A.

*Animal Studies*: N/A.

## Conflicts of Interest

The authors declare no conflicts of interest.

## Supporting information


**Data S1.** Supporting information.

**Figure S1.** Exploration of FLNA, TLN1, TIMP1, and CFL1 Gene Expression in GSE89843 and GSE68086. (A–C) Bar plots showing the comparison of FPKM values for TLN1, TIMP1, and CFL1 between NSCLC patients and healthy controls in the GSE89843 dataset. (D, F, and H) Bar plots comparing the FPKM values of TLN1, TIMP1, and CFL1 between BRC, CRC, lung cancer, hepatocellular carcinoma (HCC), pancreatic cancer, and healthy controls in the GSE68086 dataset. (E, G, I) ROC curves for TLN1, TIMP1, and CFL1 in diagnosing BRC, CRC, lung cancer, hepatocellular carcinoma, and pancreatic cancer based on the GSE68086 dataset.

**Figure S2.** Exploration of RNA concentration in serum and platelets samples. (A) Bar plots showing the comparison of RNA concentration between serum and platelets samples. (B) Bar plots showing the comparison of RNA concentration between NSCLC patients and healthy controls. (C) Bar plots showing the comparison of RNA concentration between NSCLC patients and healthy controls in serum and platelets samples, respectively. (D) Bar plots showing the comparison of RNA concentration between serum and platelets samples from NSCLC patients and healthy controls, respectively. (E) Line regression plot showing the correlation between PLT counts with RNA concentration in serum samples. Blue line indicates healthy individuals, while yellow line indicates NSCLC. (F) Line regression plot showing the correlation between WBC counts with RNA concentration in serum samples. Blue line indicates healthy individuals, while yellow line indicates NSCLC. (G) Line regression plot showing the correlation between PLT counts in whole blood samples with RNA concentration in serum samples. Blue line indicates healthy individuals, while yellow line indicates NSCLC. (H) The histogram showing the comparison of FLNA reference expression in serum and platelets from NSCLC patients and healthy controls. Serum and platelet samples from the same participant are represented by adjacent bars. Data from NSCLC patients are compared with those from healthy controls to illustrate the differences in FLNA expression levels.

**Figure S3.** The haemocytometer image of isolated platelets from 6 healthy individuals. (A–F) Haemocytometer images from 6 different healthy individuals. Each image represents one middle grid (the platelet counting area) in the haemocytometer. Red dots indicate white blood cells (WBCs), and green dots represent platelets. Due to the high platelet count, only platelets within one small grid in image (A) are annotated. Due to the limited field of view during imaging, each image is not a perfectly square shape.

**Figure S4.** Flow cytometry analysis of FLNA expression on platelets(A) Unstained cells with gating of the platelet population based on FSC and SSC. (B) Unstained cells scatter plot with APC on the x‐axis and FITC on the y‐axis. (C) Single‐staining with FLNA showing platelet scatter plot with APC on the x‐axis and FITC on the y‐axis. (D) Single‐staining with CD41 showing platelet scatter plot with APC on the x‐axis and FITC on the y‐axis. (E–H) Platelet scatter plots for four different NSCLC patients with APC on the x‐axis and FITC on the y‐axis. (I–J) Platelet scatter plots for four different healthy controls with APC on the x‐axis and FITC on the y‐axis. (M) Box plot comparing the percentage of CD41 + FLNA+ platelets between healthy controls and NSCLC patients (*p* = 0.029). (N) Linear correlation plot of the percentage of CD41 + FLNA+ platelets with peripheral blood platelet count in participants (healthy controls in red, NSCLC patients in blue).

**Figure S5.** Exploration of serum tumour marker levels in cohort 2. (A) Expression of CYFRA21‐1 and its comparison in patients with benign lung nodules, stage I, II, III, and IV NSCLC, presented as box plots. (B) Expression of NSE and its comparison in patients with benign lung nodules, stage I, II, III, and IV NSCLC, presented as box plots. (C) Expression of Pro.GRP and its comparison in patients with benign lung nodules, stage I, II, III, and IV NSCLC, presented as box plots. (D)Expression of TPS and its comparison in patients with benign lung nodules, stage I, II, III, and IV NSCLC, presented as box plots. (E) Expression of CEA and its comparison in patients with benign lung nodules, stage I, II, III, and IV NSCLC, presented as box plots. (F) Expression of FLNA and its comparison in healthy controls, benign lung nodules, LUSC, and LUAD patients, presented as box plots.

**Figure S6.** Exploration of FLNA, TLN1, TIMP1, and CFL1 Gene Expression in GSE207586. (A) Bar plots showing the comparison of FPKM values for FLNA between NSCLC patients and controls in the GSE207586 dataset. (B) Bar plots showing the comparison of FPKM values for FLNA between different stage of NSCLC patients and controls in the GSE207586 dataset. (C) Bar plots showing the comparison of FPKM values for CFL1 between NSCLC patients and controls in the GSE207586 dataset. (D) Bar plots showing the comparison of FPKM values for CFL1 between different stage of NSCLC patients and controls in the GSE207586 dataset. (E) Bar plots showing the comparison of FPKM values for TIMP1 between NSCLC patients and controls in the GSE207586 dataset. (F) Bar plots showing the comparison of FPKM values for TIMP1 between different stage of NSCLC patients and controls in the GSE207586 dataset. (G) Bar plots showing the comparison of FPKM values for TLN1 between NSCLC patients and controls in the GSE207586 dataset. (H) Bar plots showing the comparison of FPKM values for TLN1 between different stage of NSCLC patients and controls in the GSE207586 dataset.

**Figure S7.** The dianostic performance of the combined model constructing with FLNA and CYFRA 21‐1. (A) Bar plots showing the comparison of predicted values between NSCLC patients and benignant patients in the cohort2. (B) Expression of predicted values and its comparison in patients with benign lung nodules, and NSCLC, presented as box plots. (C) ROC curves for predicted values in differentiating NSCLC from benignancy based on cohort2. (D) ROC curves for predicted values in differentiating metastasis NSCLC from non‐metastasis NSCLC based on cohort2.

**Table S1.** The characteristics of individuals involved in the validation of RNA concentration in serum and platelets.

**Table S2.** The characteristics of flow cytometry cohort.

**Table S3.** Diagnostic performance of serum tumour markers and FLNA in NSCLC.

**Table S4.** An overview of logic regression showed the combination of FLNA and CYFRA21‐1.

## Data Availability

The data that support the findings of this study are available on request from the corresponding author. The data are not publicly available due to privacy or ethical restrictions.
