# Supplementary material for: The FLNA Gene in Tumour‐Educated Platelets Can Be Utilised to Identify High‐Risk Populations for NSCLCs
Source: J Cell Mol Med. 2025 Apr 10;29(7):e70544. doi: 10.1111/jcmm.70544 (PMC11984322; doi:10.1111/jcmm.70544)
Supplement: Supplementary file 1 — Data S1. Supporting information. [file JCMM-29-e70544-s003.docx]

**Supplementary Materials**

**1.** **Clinical Cohorts Description**

Cohort 1 consisted of 9 breast cancer (BRC) patients, 10 colorectal cancer (CRC) patients, 17 healthy controls, and 18 NSCLC patients (Table 1 and Table2). This cohort was used to explore FLNA expression in platelets from different cancer patients and to compare FLNA expression between serum and platelets. The detailed information of the samples in this cohort is provided in Supplementary Table 5.

Cohort 2 included 48 NSCLC patients, 29 benign patients, and 39 healthy controls. The primary aim of this cohort was to investigate FLNA expression in platelets from NSCLC patients and benign lung nodule patients, as well as to assess the potential value of platelet FLNA expression for predicting NSCLC onset and prognosis. Diagnostic comparisons were made with common serum tumor markers. The detailed information of the samples in this cohort is provided in Supplementary Table 6.

Cohort 3 included 4 NSCLC patients and 4 healthy controls, and was specifically designed for flow cytometry analysis to confirm FLNA expression in platelets from NSCLC patients. The detailed information of the samples in this cohort is provided in Supplementary Table 7.

Cohort 4 consisted of 16 healthy individuals and 9 NSCLC patients. This cohort was used to explore differences in RNA concentrations between serum and platelets, as well as to assess the purity of platelet samples. The detailed information of the samples in this cohort is provided in Supplementary Table 8.

**2. Exploration of FLNA, TLN1, TIMP1, and CFL1 Gene Expression in GSE68086 and GSE207586**

To explore the expression of FLNA, TLN1, TIMP1, and CFL1 in non-small cell lung cancer (NSCLC), we analyzed two publicly available datasets: GSE68086 and GSE207586. Both datasets contain gene expression profiles from NSCLC patients and healthy controls.

**2.1 Dataset Information**

**GSE68086**: An RNA sequencing data for 283 platelet samples with six different malignant tumors (NSCLC, CRC, PCA, GBM, BrCA, and HC) (https://www.ncbi.nlm.nih.gov/geo/).

**GSE207586**: This dataset is also RNA-seq data of 766 blood platelet samples, including 399 Non-Small Cell Lung Cancer (NSCLC) patients from different stages (I-IV) and 367 asymptomatic individuals (Controls) (https://www.ncbi.nlm.nih.gov/geo/).

**2.2 Methods**

We used **FPKM** (Fragments Per Kilobase of transcript per Million mapped reads) values to standardize gene expression levels across samples. Expression levels of FLNA, TLN1, TIMP1, and CFL1 were compared between NSCLC patients and healthy controls using t-tests to assess statistical significance. Receiver Operating Characteristic (ROC) curves were generated for FLNA, TLN1, TIMP1, and CFL1 to evaluate their diagnostic performance in distinguishing NSCLC from healthy individuals.

**2.3 Results**

In both datasets, TLN1, TIMP1, and CFL1 were expressed at higher levels in NSCLC patients compared to healthy controls (Supplementary figure 1, Supplementary Figure 6). FLNA expression was significantly higher in NSCLC patients compared to healthy individuals in GSE68086, but no significant difference was observed in GSE207586 (Supplementary Figure 6). In GSE207586, a comparison of FPKM values between different stages of NSCLC and non-NSCLC revealed a possible difference in Stage I and non-NSCLC (p=0.076), suggesting that FLNA may serve as a potential biomarker for predicting early-stage NSCLC compared to healthy individuals. ROC curve analysis revealed that FLNA demonstrated better diagnostic performance for distinguishing NSCLC from healthy controls compared to TLN1, TIMP1, and CFL1 in both datasets (Fig 4, Supplementary figure 1).

Interestingly, although CFL1, TIMP1, and TLN1 showed significant differences between NSCLC and control groups in GSE207586, no significant differences were observed across different stages of NSCLC. CFL1 and TIMP1 exhibited significant differences between Stage IV and non-NSCLC (p=0.00077, p=0.0042), while TLN1 showed no significant differences across any stages (p>0.1) (Supplementary Figure 6). These findings suggest that these three genes may have limited value in the early diagnosis of NSCLC.

**2.4 Conclusion**

### FLNA demonstrates superior diagnostic performance for NSCLC compared to other genes such as TLN1, TIMP1, and CFL1. In both datasets, TLN1, TIMP1, and CFL1 were upregulated in NSCLC compared to healthy controls, but FLNA exhibited more consistent results. While FLNA was significantly higher in NSCLC patients in GSE68086, it showed a possible early-stage difference in GSE207586 (p=0.076), suggesting its potential as a biomarker for early-stage NSCLC. ROC curve analysis confirmed that FLNA outperformed TLN1, TIMP1, and CFL1 in distinguishing NSCLC from healthy controls. Interestingly, CFL1, TIMP1, and TLN1 showed no significant stage-dependent differences in NSCLC, with only CFL1 and TIMP1 distinguishing Stage IV from non-NSCLC. These findings suggest that FLNA is more reliable for early-stage diagnosis, while the other genes have limited diagnostic value.

### 3.Validation of RNA Concentration in Serum and Platelets in Cohort 4

To validate the RNA concentrations in serum and platelets, we collected data from a separate clinical cohort (Cohort 4), which included 16 healthy individuals and 9 NSCLC patients. The primary objective was to exclude the potential confounding effects of platelets and leukocytes in serum on RNA concentration.

**3.1** **Methods**

**Blood Cell Count**: We utilized a Mindray BC5390 Hematology Analyzer to measure platelet and white blood cell (WBC) counts in the serum samples, ensuring that the presence of these cells did not affect RNA concentration measurements.

**Platelet Purity**: Platelets were isolated from peripheral blood by centrifugation, and the purity of the platelet preparations was verified using a hemocytometer. Microscopic examination confirmed the absence of contaminating leukocytes and erythrocytes.

**RNA Extraction and Comparison**: RNA was extracted from both serum and platelet samples using the same RNA extraction kit for consistency. The RNA concentrations were compared between serum and platelets to evaluate potential differences.

**Statistical Analysis**: Linear correlation analysis was performed to determine if RNA concentrations in serum were influenced by platelet and WBC counts. Additionally, GAPDH was used as the reference gene for relative expression quantification in both serum and platelet samples.

**3.2** **Results**

**RNA Concentration**: The results showed that RNA concentrations in platelets were significantly higher than those in serum samples. However, the RNA concentration in serum did not correlate with platelet or WBC counts (Supplementary figure2).

**Platelet Purity**: To confirm the purity of platelet isolation, we utilized a hemocytometer for direct counting of platelets and leukocytes (WBCs) under a microscope (Supplementary Figure3). The hemocytometer image shows the following:

- Red Dots: These are marked as WBCs (White Blood Cells), which were clearly distinguishable due to their larger size and irregular shape compared to platelets.
- Green Dots: These are marked as platelets, which are smaller and more numerous. Due to the high platelet count, we only annotated the platelets within one small grid area (as shown in the first image) (Supplementary Figure3A) to avoid overcrowding and ensure accurate counting.
- This method effectively allowed us to assess the purity of the platelet preparation, confirming the absence of significant contamination by leukocytes or erythrocytes.

**Impact of RNA Concentration on Relative Expression**: To minimize potential errors caused by RNA concentration differences, we calculated the relative expression of FLNA in serum and platelet samples using GAPDH as the reference gene.

**3.3 Conclusion**

The RNA concentration in serum was lower than in platelets, but there was no correlation between serum RNA levels and the blood cell counts (platelet and WBC) (Supplementary figure2). This suggests that the differences in RNA concentrations are not due to the presence of platelets or leukocytes in the serum. Therefore, for subsequent analyses, relative expression levels were calculated separately for serum and platelet groups using GAPDH, which may help mitigate the influence of RNA concentration variations.

**4. Exploration of FLNA Expression in Cohort 1**

To further investigate FLNA expression in various groups, we analyzed **Cohort 1**, which consisted of 9 breast cancer (BRC) patients, 10 colorectal cancer (CRC) patients, 17 healthy controls, and 18 NSCLC patients (Table 1). The details of sample collection were as follows:

**Healthy Controls**: Blood samples from 17 healthy individuals were collected, including 7 platelet (PLT) samples and 17 serum samples.

**NSCLC Patients**: Blood samples from 18 NSCLC patients were collected, including 13 platelet (PLT) samples and 18 serum samples.

**BRC and CRC Patients**: Only platelet (PLT) samples were collected from 9 BRC and 10 CRC patients.

### ****4.1 FLNA Expression in Platelets of NSCLC vs. Other Cancers****

We first compared FLNA expression in platelets from 13 NSCLC patients and 7 healthy controls, as well as 9 BRC and 10 CRC patients, to investigate whether FLNA expression in platelets is elevated in NSCLC and potentially specific to this cancer type (**Fig 3D**). Our findings suggest that FLNA expression in platelets from NSCLC patients was significantly higher than that in other cancer types, such as BRC and CRC, highlighting its potential as a tumor-specific biomarker for NSCLC.

### ****4.2 FLNA Expression in Serum vs. Platelets****

Next, we analyzed FLNA expression in serum and platelets for further investigation of its potential as a TEP biomarker. FLNA expression was measured in serum and platelets from 17 healthy individuals (7 platelet and 17 serum samples) and 18 NSCLC patients (13 platelet and 18 serum samples) (**Fig 3E, F; Supplementary figure 2H**). The comparison revealed that FLNA expression was significantly higher in platelets than in serum, supporting the notion that FLNA expression in platelets could be a more reliable marker in NSCLC and potentially indicative of TEP-specific changes.

**5.** **Flow Cytometry Analysis of FLNA Expression**

To further investigate FLNA expression on platelets in NSCLC, flow cytometry was employed. Platelet samples from 4 NSCLC patients, 4 healthy controls were analyzed (clinical cohort 3). The expression of FLNA on platelets was quantified using the flow cytometer setup described below:

**5.1 Methods**

**5.1.1 Sample Preparation**

10 μl of platelet-rich plasma (PRP) was added to the bottom of a flow cytometry tube. 500 μl of pre-cooled (4°C) fixative solution was added and incubated at room temperature for 10 minutes. The sample was then centrifuged at 1000 rpm for 10 minutes. After discarding the supernatant, 1 wash with PBS was performed, followed by another centrifugation at 1000 rpm for 10 minutes and discarding the supernatant.

**5.1.2 Cell Permeabilization**

1.5 ml of permeabilization solution was added, and the sample was incubated at room temperature for 10-15 minutes. After centrifugation at 1000 rpm for 5 minutes, the supernatant was discarded, and cells were resuspended in an appropriate volume of permeabilization solution.

**5.1.3 Antibody Staining**

2 μl of APC-conjugated anti-human CD41a antibody (BD Pharmingen, Cat 559777) and 2 μl of FITC-conjugated rabbit anti-human Filamin A antibody (Abcam, Alexa Fluor® 488 Anti-Filamin A [EP2405Y], ab246749) were added to the tube. Separate tubes were prepared for single staining with CD41a and Filamin A antibodies. The tubes were vortexed and incubated at room temperature in the dark for 20 minutes.

**5.1.4 Washing**

After incubation, PBS was added, followed by centrifugation at 1000 rpm for 5 minutes. The supernatant was discarded, and cells were resuspended in 200 μl of PBS.

**5.1.5 Flow Cytometry Analysis**

The samples were analyzed using the Cytek® Aurora/Northern Lights™ full-spectrum flow cytometer as soon as possible after preparation. Fixed samples should be analyzed within 48 hours.

#### 5.1.6 Flow Cytometry Data Acquisition and Analysis

#### ****Instrument Setup****: The full-spectrum flow cytometry software was used to acquire and analyze data. The settings for FSC and SSC were adjusted, with APC fluorescence-labeled CD41a as the platelet marker. Voltage adjustments were made to minimize cell debris and instrument background noise, with a low flow rate used to reduce cell aggregation.

**Gating Strategy**: Data were collected from unstained controls and single-staining control tubes. Gating was applied on the scatter plots of the unstained and platelet single-staining tubes to define the population of platelets. The positive gate for CD41a-APC/SSC was drawn to identify platelets, ensuring that platelet clusters were positioned at the highest intensity in the R1 channel of the scatter plot. The FITC-conjugated Filamin A antibody-positive population was gated within the R1 channel.

**Data Collection**: Data from control and experimental tubes were collected, with a minimum of 10^4^ platelets recorded per sample. The percentage of FLNA-positive platelets was determined by analyzing the data from the APC and FITC channels, representing the level of FLNA expression on platelets. Data analysis was performed using FlowJo software.

**5.2 Results**

**5.2.1** **Basic information of Flow Cytometry cohort**

The basic information of cohort 3 including 4 healthy individuals and 4 NSCLC patients in the flow cytometry analysis is summarized in **Supplementary table2**. There were no significant differences between the two groups in terms of gender, age, or platelet parameters.

**Supplementary table2.** **The Characteristics of Flow Cytometry cohort**

|  | **level** | **healthy** | **malignancy** | **p** | **test** |
| --- | --- | --- | --- | --- | --- |
| **n** |  | 4 | 4 |  |  |
| **Stage** | healthy | 4 (100.0) | 0 (0.0) | 0.046 |  |
|  | I | 0 (0.0) | 2 (50.0) |  |  |
|  | III | 0 (0.0) | 1 (25.0) |  |  |
|  | IV | 0 (0.0) | 1 (25.0) |  |  |
| **Gender** | 0 | 1 (25.0) | 2 (50.0) | 1 |  |
|  | 1 | 3 (75.0) | 2 (50.0) |  |  |
| **Pathology** | healthy | 4 (100.0) | 0 (0.0) | 0.018 |  |
|  | LUAD | 0 (0.0) | 3 (75.0) |  |  |
|  | LUSC | 0 (0.0) | 1 (25.0) |  |  |
| **Metastasis** | 0 | 0 (NaN) | 2 (50.0) | NaN |  |
|  | 1 | 0 (NaN) | 2 (50.0) |  |  |
| **age (median,IQR)** |  | 45.50 [34.25, 58.00] | 61.50 [49.00, 71.00] | 0.245 | nonnorm |
| **PLT(×10^9/L, median, IQR)** |  | 201.50 [159.50, 237.50] | 219.50 [172.25, 300.00] | 0.663 | nonnorm |
| **MPV(fL, median, IQR)** |  | 12.00 [11.30, 12.38] | 11.00 [9.85, 12.22] | 0.885 | nonnorm |
| **PDW(median,IQR)** |  | 16.65 [16.42, 16.75] | 16.40 [16.38, 16.42] | 0.245 | nonnorm |
| **PCT(%, median,IQR)** |  | 0.22 [0.20, 0.24] | 0.24 [0.18, 0.34] | 0.773 | nonnorm |
| **CD41-FLNA+PLT (%, median, IQR)** |  | 5.76 [0.02, 12.80] | 3.91 [2.62, 5.22] | 0.773 | nonnorm |
| **CD41+FLNA+PLT (%, median, IQR)** |  | 82.95 [80.28, 84.12] | 93.55 [92.70, 94.80] | 0.021 | nonnorm |
| **CD41+FLNA-PLT (%, median, IQR)** |  | 9.43 [4.62, 14.42] | 1.29 [0.58, 1.97] | 0.021 | nonnorm |
| **CD41-FLNA-PLT (%, median, IQR)** |  | 1.48 [0.00, 3.34] | 1.09 [0.72, 1.26] | 0.767 | nonnorm |

**5.2.2 Comparison of FLNA expression on platelets between healthy and NSCLC**

**Supplementary Figure 4** shows representative flow cytometry plots illustrating FLNA expression on platelets from NSCLC patients and controls. Scatter plots of the unstained control and platelet single-staining tubes are shown, along with the gating process. Following gating, scatter plots for the 4 healthy individuals and 4 NSCLC patients are presented.

Additionally, the percentage of CD41+FLNA+ double-positive cells was compared between the two groups, and it was found that NSCLC patients exhibited a significantly higher percentage of CD41+FLNA+ double-positive cells compared to healthy controls. Linear analysis revealed that, in healthy individuals, the percentage of CD41+FLNA+ double-positive cells was potentially correlated with the total platelet count in peripheral blood. However, no such correlation was observed in NSCLC patients.

**5.3 Conclusion**

Although the small sample size limits the generalizability of the results, the observed trend suggests that FLNA expression in platelets may be elevated in NSCLC patients. Further studies with larger sample sizes are needed to validate these findings. Due to the limited sample size, this section of the study was presented in the supplementary materials rather than the main part of the manuscript.

**6.** **Exploring the** **diagnostic value of FLNA in combination with other serum tumor markers**

**6.1 Methods**

To evaluate the combined diagnostic value of FLNA and other serum tumor markers (Pro-GRP, NSE, CEA, TPS, and CYFRA 21-1), we performed logistic regression analysis. Initially, all five markers were included in the model, and based on the p-values, we systematically removed markers that were not significantly associated with the outcome. After this process, only CYFRA 21-1 and FLNA were retained for further analysis.

The diagnostic performance of the combined model was assessed by calculating the AUC from ROC curve analysis, and we examined the differences in predictive values between NSCLC patients and benign lung nodules, as well as across different NSCLC stages.

**6.2 Results**

**6.2.1** The final formula for the combined predictive value was determined as follows:

Predictive Value=-3.1248+ 1.2117* FLNa + 0.8294* CYFRA21-1

**Supplementary Table 4 An overview of logic regression showed the combination of FLNA and CYFRA21-1**

|  | **Estimate** | **Std. Error** | **Z value** | **P** |
| --- | --- | --- | --- | --- |
| **(Intercept)** | -3.1248 | 1.1810 | -2.646 | 0.00815 |
| **FLNa** | 1.2117 | 0.7070 | 1.714 | 0.08656 |
| **CYFRA21-1** | 0.8294 | 0.3496 | 2.373 | 0.01766 |

**6.2.2 The diagnostic value of Predictive Value**

After performing the logistic regression, CYFRA 21-1 and FLNA were retained as significant predictors. The predictive value combining CYFRA 21-1 and FLNA was calculated for each participant (Supplementary figure 7 A, B). The ROC curve analysis demonstrated that the combined predictive value had an AUC of 0.839 (95% CI: 0.733-0.945) for distinguishing NSCLC from benign lung nodules (Supplementary figure 7 C), and an AUC of 0.709 (95% CI: 0.533- 0.885) for distinguishing metastatic from non-metastatic NSCLC (Supplementary figure 7 D). These results suggest that the combined model provides a reliable diagnostic tool for distinguishing NSCLC from benign lung nodules and for evaluating the metastatic status of NSCLC patients.

**Conclusion**

The development of a combined marker requires a rigorous modeling and validation cohort. However, due to the limited sample size in this study, the constructed model may not be fully reliable, and further validation with larger sample sizes is needed. Nonetheless, our findings indicate that FLNA, when combined with common tumor markers, shows promise in distinguishing different stages of NSCLC and can compensate for FLNA's limitations in diagnostic accuracy. In future research, we plan to expand the sample size and further refine this study.

**7. Supplementary table legends**

Supplementary Table 1. The Characteristics of individuals involved in the validation of RNA concentration in serum and platelets

Supplementary Table 2. The Characteristics of Flow Cytometry cohort

Supplementary Table 3. Diagnostic Performance of Serum Tumor Markers and FLNA in NSCLC

Supplementary Table 4. An overview of logic regression showed the combination of FLNA and CYFRA21-1

Supplementary Table 5. Detailed Information of cohort 1

Supplementary Table 6. Detailed Information of cohort 2

Supplementary Table 7. Detailed Information of cohort 3 (Flow Cytometry cohort)

Supplementary Table 8. Detailed Information of cohort 4 (validation cohort of RNA concentration in serum and platelets)

**8. Supplementary Figure legends**

**Supplementary Figure1.** **Exploration of FLNA, TLN1, TIMP1, and CFL1 Gene Expression in GSE89843 and GSE68086. (A–C)** Bar plots showing the comparison of FPKM values for TLN1, TIMP1, and CFL1 between NSCLC patients and healthy controls in the GSE89843 dataset. **(D, F, H)** Bar plots comparing the FPKM values of TLN1, TIMP1, and CFL1 between BRC, CRC, lung cancer, hepatocellular carcinoma (HCC), pancreatic cancer, and healthy controls in the GSE68086 dataset. **(E, G, I)** ROC curves for TLN1, TIMP1, and CFL1 in diagnosing BRC, CRC, lung cancer, hepatocellular carcinoma, and pancreatic cancer based on the GSE68086 dataset.

**Supplementary Figure2. Exploration of** **RNA concentration in s****erum and platelets samples.** **(A)** Bar plots showing the comparison of RNA concentration between serum and platelets samples. **(B)** Bar plots showing the comparison of RNA concentration between NSCLC patients and healthy controls. **(C)** Bar plots showing the comparison of RNA concentration between NSCLC patients and healthy controls in serum and platelets samples, respectively. **(D)** Bar plots showing the comparison of RNA concentration between serum and platelets samples from NSCLC patients and healthy controls, respectively. **(E)** Line regression plot showing the correlation between PLT counts with RNA concentration in serum samples. Blue line indicates healthy individuals, while yellow line indicates NSCLC. **(F)** Line regression plot showing the correlation between WBC counts with RNA concentration in serum samples. Blue line indicates healthy individuals, while yellow line indicates NSCLC. **(G)** Line regression plot showing the correlation between PLT counts in whole blood samples with RNA concentration in serum samples. Blue line indicates healthy individuals, while yellow line indicates NSCLC. **(H)** The histogram showing the comparison of FLNA reference expression in serum and platelets from NSCLC patients and healthy controls. Serum and platelet samples from the same participant are represented by adjacent bars. Data from NSCLC patients are compared with those from healthy controls to illustrate the differences in FLNA expression levels.

**Supplementary Figure3. The hemocytometer image of isolated platelets from 6 healthy individuals. (A–F)** Hemocytometer images from 6 different healthy individuals. Each image represents one middle grid (the platelet counting area) in the hemocytometer. Red dots indicate white blood cells (WBCs), and green dots represent platelets. Due to the high platelet count, only platelets within one small grid in image (A) are annotated. Due to the limited field of view during imaging, each image is not a perfectly square shape.

**Supplementary Figure4. Flow Cytometry Analysis of FLNA Expression on Platelets**

**(A)** Unstained cells with gating of the platelet population based on FSC and SSC. **(B)** Unstained cells scatter plot with APC on the x-axis and FITC on the y-axis. **(C)** Single-staining with FLNA showing platelet scatter plot with APC on the x-axis and FITC on the y-axis. **(D)** Single-staining with CD41 showing platelet scatter plot with APC on the x-axis and FITC on the y-axis. **(E–H)** Platelet scatter plots for four different NSCLC patients with APC on the x-axis and FITC on the y-axis. **(I–J)** Platelet scatter plots for four different healthy controls with APC on the x-axis and FITC on the y-axis. **(M)** Box plot comparing the percentage of CD41+FLNA+ platelets between healthy controls and NSCLC patients (p=0.029). **(N)** Linear correlation plot of the percentage of CD41+FLNA+ platelets with peripheral blood platelet count in participants (healthy controls in red, NSCLC patients in blue).

**Supplementary Figure5.** **Exploration of serum tumor marker levels in cohort 2. (A)** Expression of CYFRA21-1 and its comparison in patients with benign lung nodules, stage I, II, III, and IV NSCLC, presented as box plots. **(B)** Expression of NSE and its comparison in patients with benign lung nodules, stage I, II, III, and IV NSCLC, presented as box plots. **(C)** Expression of Pro.GRP and its comparison in patients with benign lung nodules, stage I, II, III, and IV NSCLC, presented as box plots. **(D)**Expression of TPS and its comparison in patients with benign lung nodules, stage I, II, III, and IV NSCLC, presented as box plots. **(E)** Expression of CEA and its comparison in patients with benign lung nodules, stage I, II, III, and IV NSCLC, presented as box plots. **(F)** Expression of FLNA and its comparison in healthy controls, benign lung nodules, LUSC, and LUAD patients, presented as box plots.

**Supplementary Figure 6. Exploration of** **FLNA, TLN1, TIMP1, and CFL1 Gene Expression in** **GSE****207586.** **(A)** Bar plots showing the comparison of FPKM values for FLNA between NSCLC patients and controls in the GSE207586 dataset. **(B)** Bar plots showing the comparison of FPKM values for FLNA between different stage of NSCLC patients and controls in the GSE207586 dataset. **(C)**Bar plots showing the comparison of FPKM values for CFL1 between NSCLC patients and controls in the GSE207586 dataset. **(D)** Bar plots showing the comparison of FPKM values for CFL1 between different stage of NSCLC patients and controls in the GSE207586 dataset. **(E)** Bar plots showing the comparison of FPKM values for TIMP1 between NSCLC patients and controls in the GSE207586 dataset. **(F)** Bar plots showing the comparison of FPKM values for TIMP1 between different stage of NSCLC patients and controls in the GSE207586 dataset. **(G)** Bar plots showing the comparison of FPKM values for TLN1 between NSCLC patients and controls in the GSE207586 dataset. **(H)** Bar plots showing the comparison of FPKM values for TLN1 between different stage of NSCLC patients and controls in the GSE207586 dataset.

**Supplementary Figure 7. The dianostic performance of the combined model constructing with FLNA and CYFRA 21-1. (A)** Bar plots showing the comparison of predicted values between NSCLC patients and benignant patients in the cohort2. **(B)** Expression of predicted values and its comparison in patients with benign lung nodules, and NSCLC, presented as box plots. **(C)** ROC curves for predicted values in differentiating NSCLC from benignancy based on cohort2. **(D)** ROC curves for predicted values in differentiating metastasis NSCLC from non-metastasis NSCLC based on cohort2.
