# Supplementary figures and images for: The FLNA Gene in Tumour‐Educated Platelets Can Be Utilised to Identify High‐Risk Populations for NSCLCs
Source: J Cell Mol Med. 2025 Apr 10;29(7):e70544. doi: 10.1111/jcmm.70544 (PMC11984322; doi:10.1111/jcmm.70544)

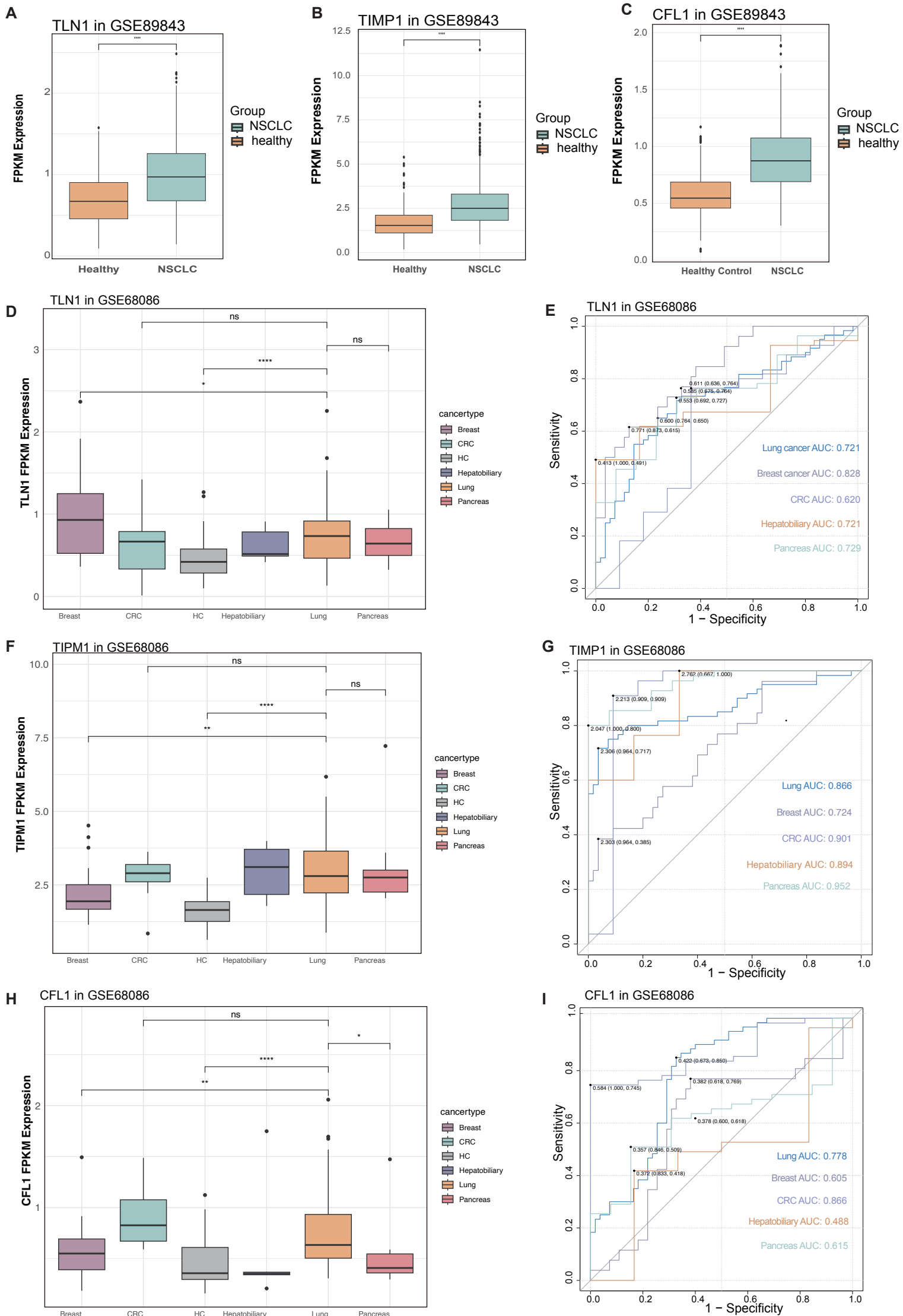

Supplement: Supplementary file 2 — Figure S1. Exploration of FLNA, TLN1, TIMP1, and CFL1 Gene Expression in GSE89843 and GSE68086. (A–C) Bar plots showing the comparison of FPKM values for TLN1, TIMP1, and CFL1 between NSCLC patients and healthy controls in the GSE89843 dataset. (D, F, and H) Bar plots comparing the FPKM values of TLN1, TIMP1, and CFL1 between BRC, CRC, lung cancer, hepatocellular carcinoma (HCC), pancreatic cancer, and healthy controls in the GSE68086 dataset. (E, G, I) ROC curves for TLN1, TIMP1, and CFL1 in diagnosing BRC, CRC, lung cancer, hepatocellular carcinoma, and pancreatic cancer based on the GSE68086 dataset. [file JCMM-29-e70544-s002.pdf]

**A**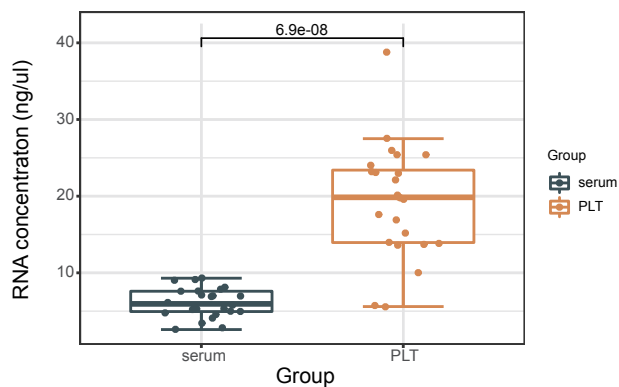**B**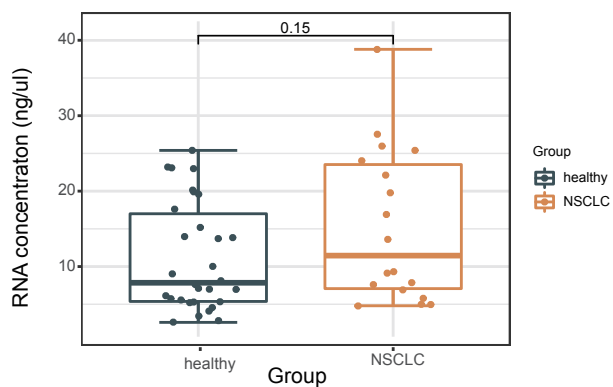**C**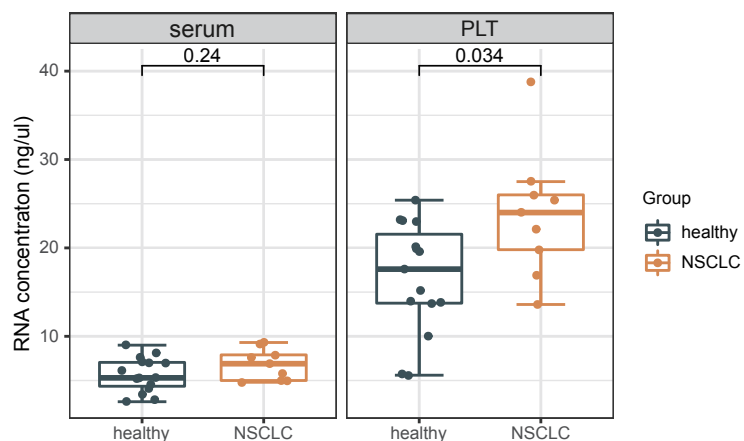**D**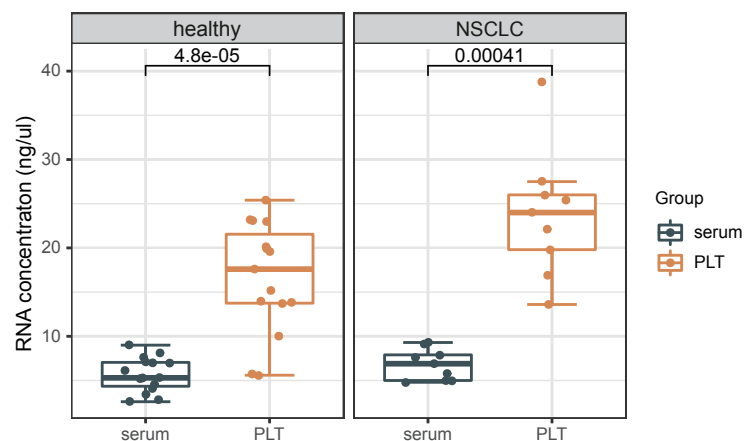**E**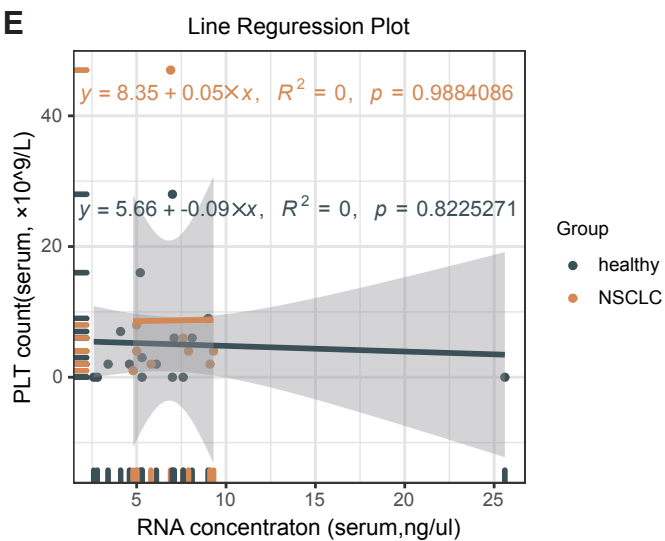**F**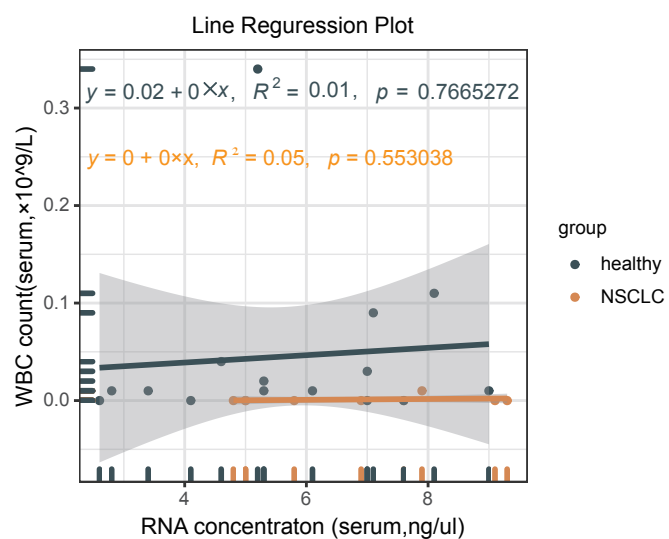**G**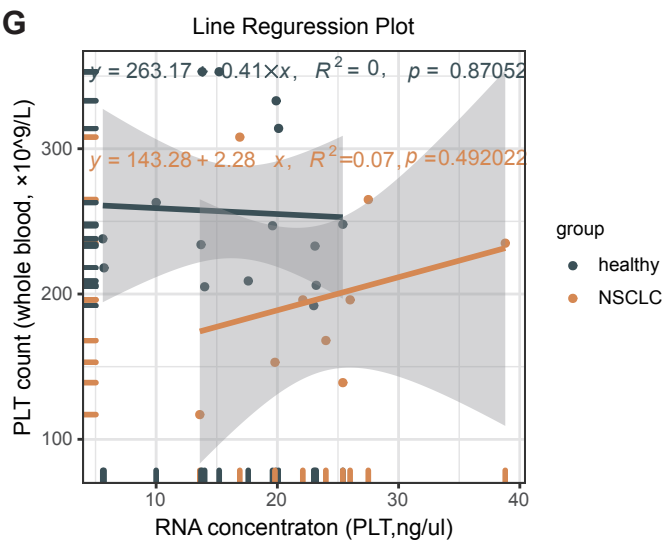**H**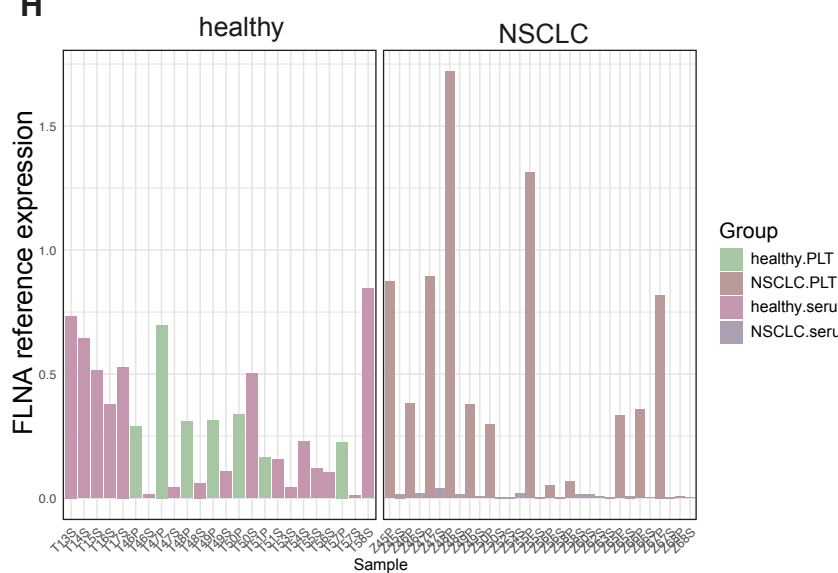

Supplement: Supplementary file 3 — Figure S2. Exploration of RNA concentration in serum and platelets samples. (A) Bar plots showing the comparison of RNA concentration between serum and platelets samples. (B) Bar plots showing the comparison of RNA concentration between NSCLC patients and healthy controls. (C) Bar plots showing the comparison of RNA concentration between NSCLC patients and healthy controls in serum and platelets samples, respectively. (D) Bar plots showing the comparison of RNA concentration between serum and platelets samples from NSCLC patients and healthy controls, respectively. (E) Line regression plot showing the correlation between PLT counts with RNA concentration in serum samples. Blue line indicates healthy individuals, while yellow line indicates NSCLC. (F) Line regression plot showing the correlation between WBC counts with RNA concentration in serum samples. Blue line indicates healthy individuals, while yellow line indicates NSCLC. (G) Line regression plot showing the correlation between PLT counts in whole blood samples with RNA concentration in serum samples. Blue line indicates healthy individuals, while yellow line indicates NSCLC. (H) The histogram showing the comparison of FLNA reference expression in serum and platelets from NSCLC patients and healthy controls. Serum and platelet samples from the same participant are represented by adjacent bars. Data from NSCLC patients are compared with those from healthy controls to illustrate the differences in FLNA expression levels. [file JCMM-29-e70544-s009.pdf]

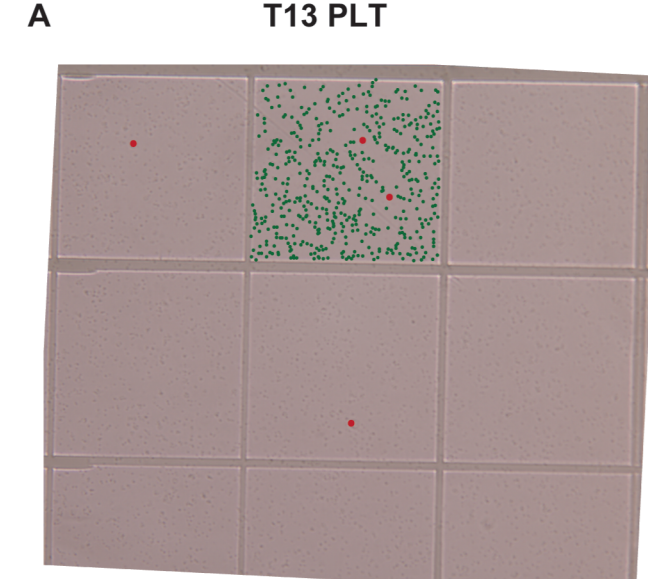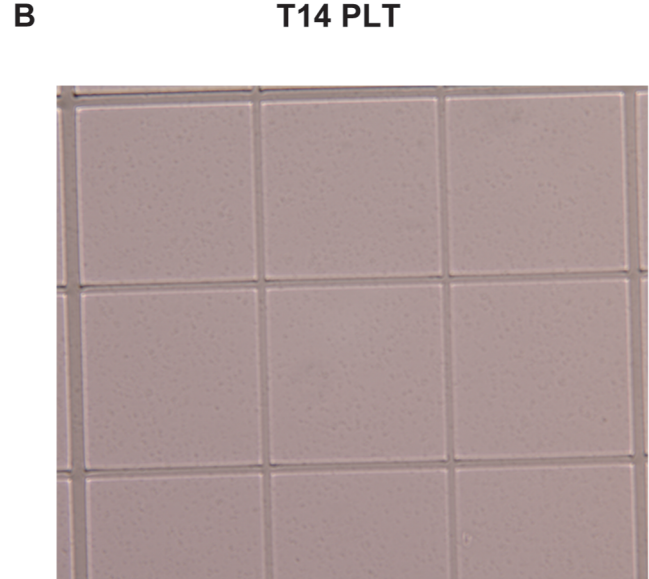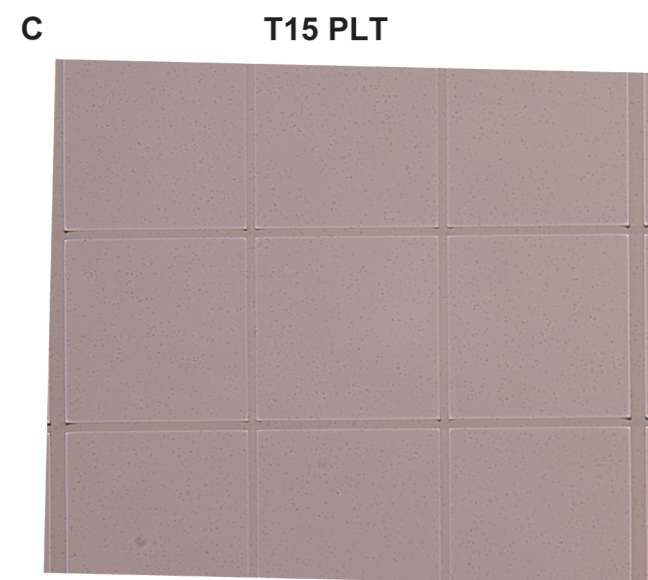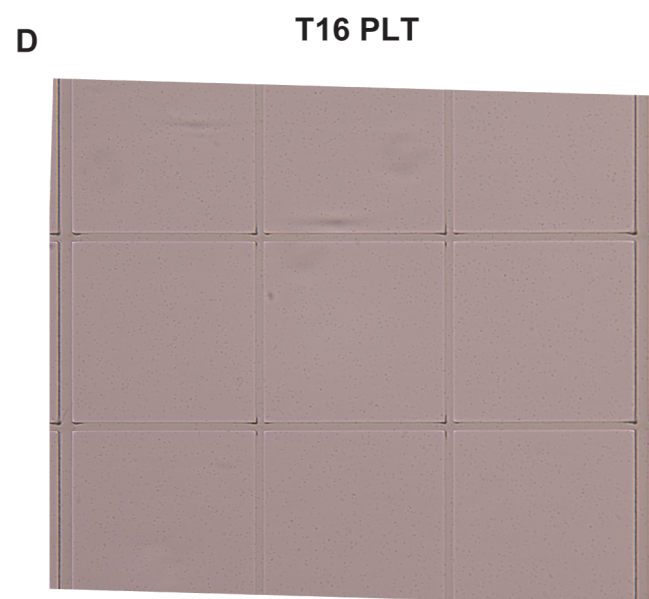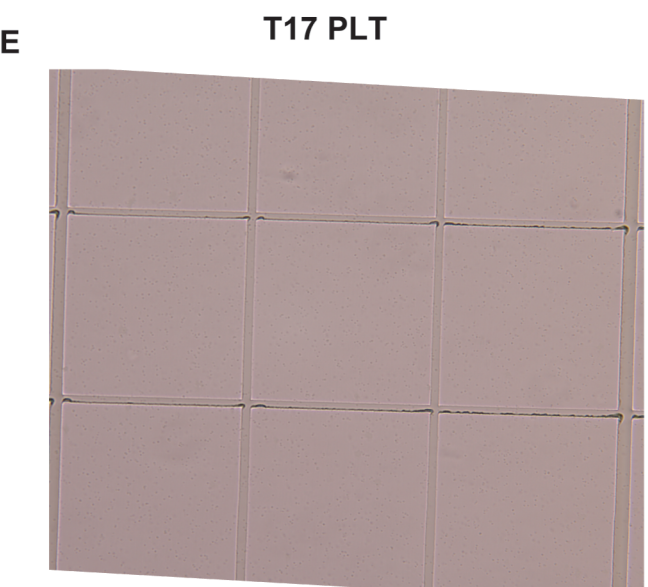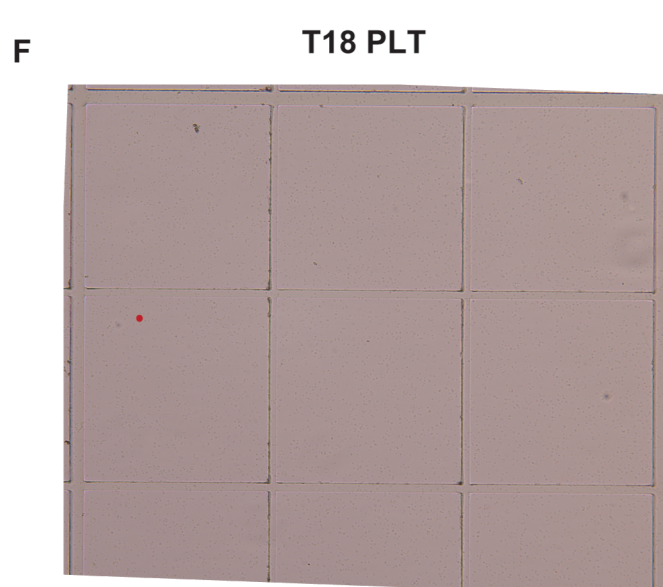

Supplement: Supplementary file 4 — Figure S3. The haemocytometer image of isolated platelets from 6 healthy individuals. (A–F) Haemocytometer images from 6 different healthy individuals. Each image represents one middle grid (the platelet counting area) in the haemocytometer. Red dots indicate white blood cells (WBCs), and green dots represent platelets. Due to the high platelet count, only platelets within one small grid in image (A) are annotated. Due to the limited field of view during imaging, each image is not a perfectly square shape. [file JCMM-29-e70544-s004.pdf]

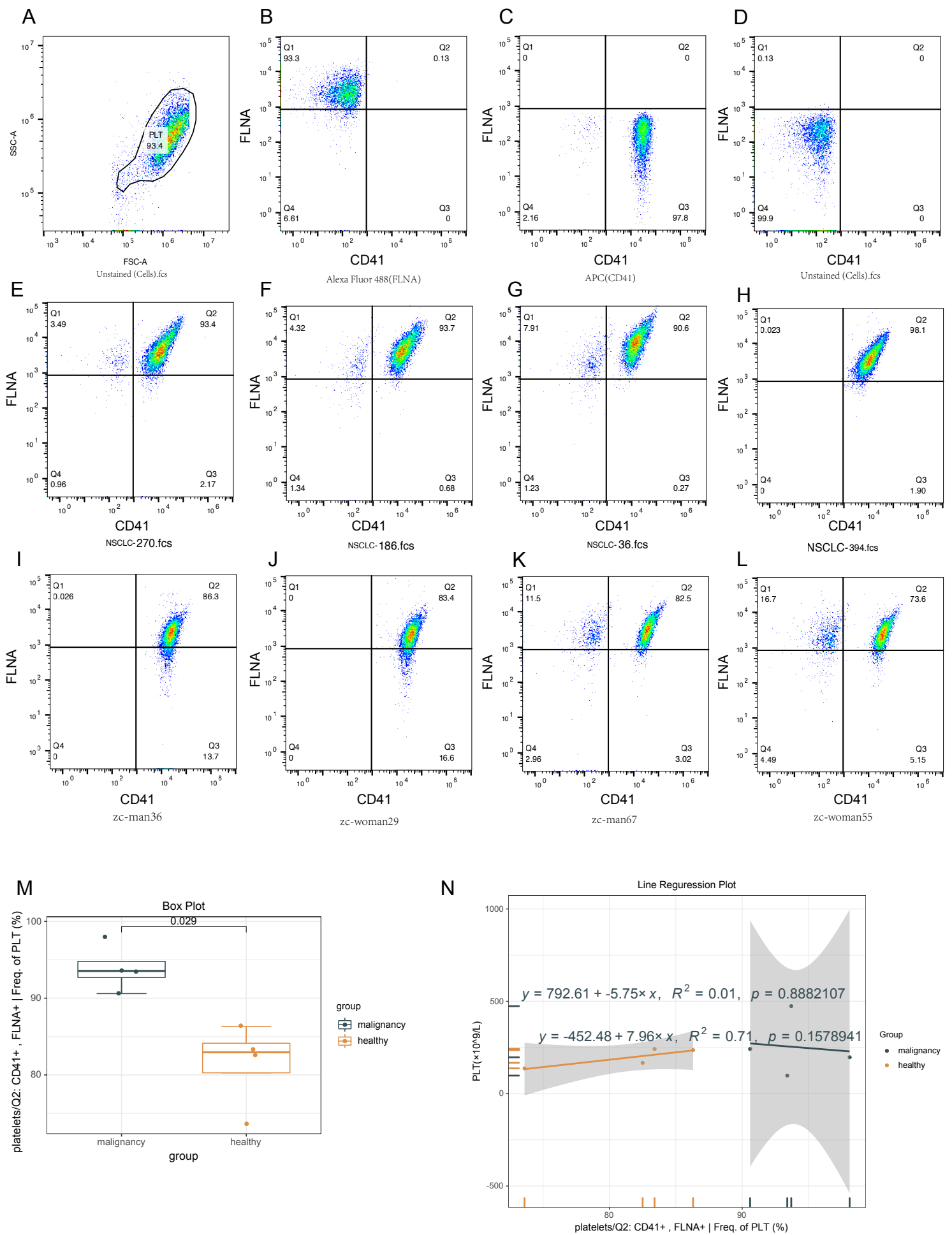

Supplement: Supplementary file 5 — Figure S4. Flow cytometry analysis of FLNA expression on platelets(A) Unstained cells with gating of the platelet population based on FSC and SSC. (B) Unstained cells scatter plot with APC on the x‐axis and FITC on the y‐axis. (C) Single‐staining with FLNA showing platelet scatter plot with APC on the x‐axis and FITC on the y‐axis. (D) Single‐staining with CD41 showing platelet scatter plot with APC on the x‐axis and FITC on the y‐axis. (E–H) Platelet scatter plots for four different NSCLC patients with APC on the x‐axis and FITC on the y‐axis. (I–J) Platelet scatter plots for four different healthy controls with APC on the x‐axis and FITC on the y‐axis. (M) Box plot comparing the percentage of CD41 + FLNA+ platelets between healthy controls and NSCLC patients (p = 0.029). (N) Linear correlation plot of the percentage of CD41 + FLNA+ platelets with peripheral blood platelet count in participants (healthy controls in red, NSCLC patients in blue). [file JCMM-29-e70544-s006.pdf]

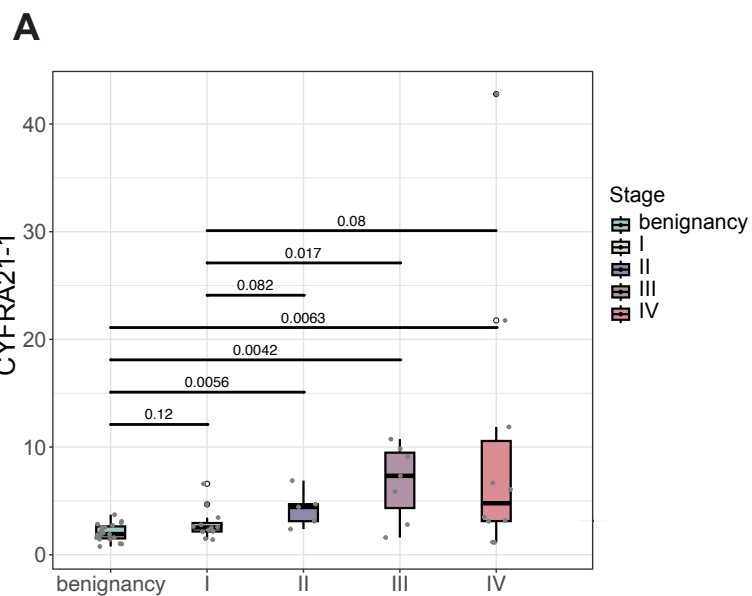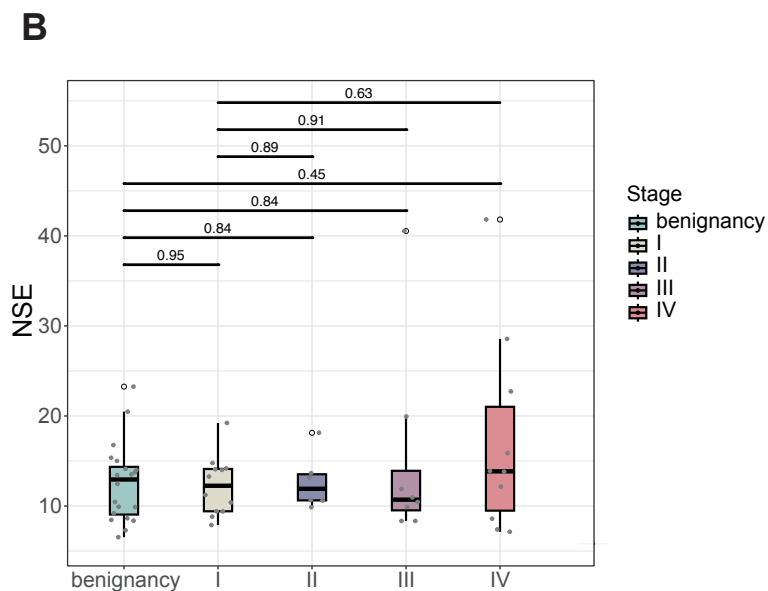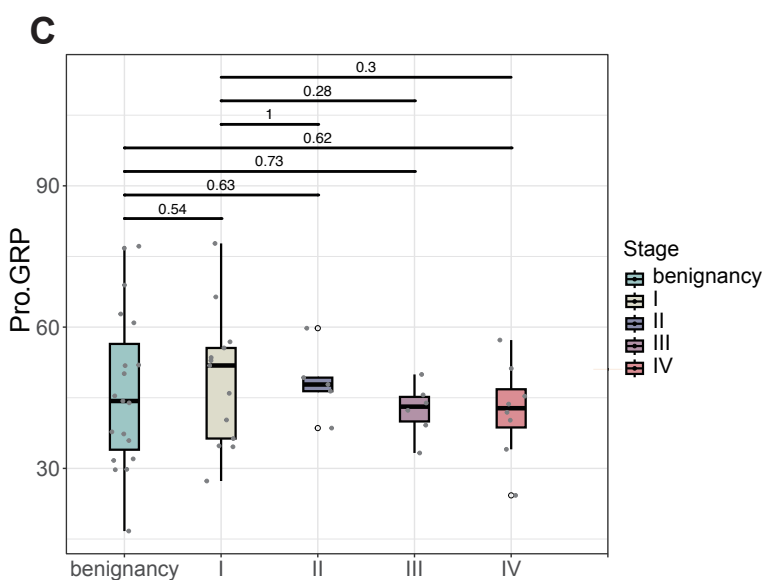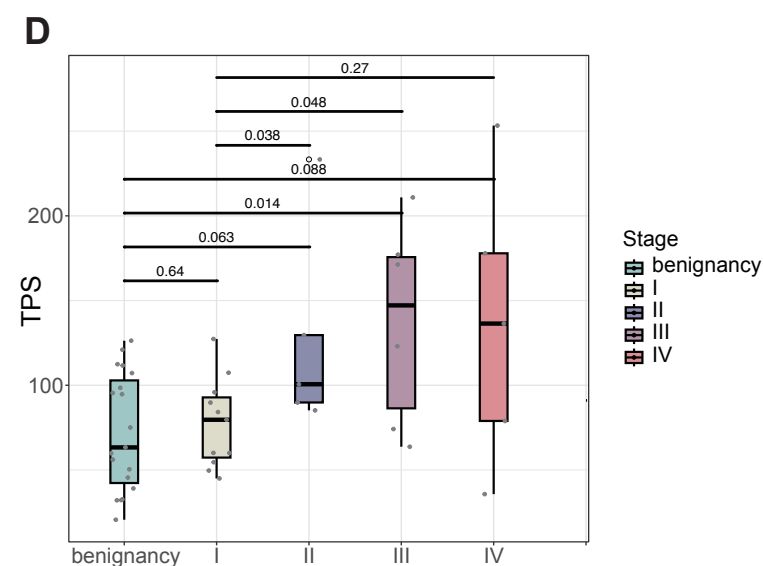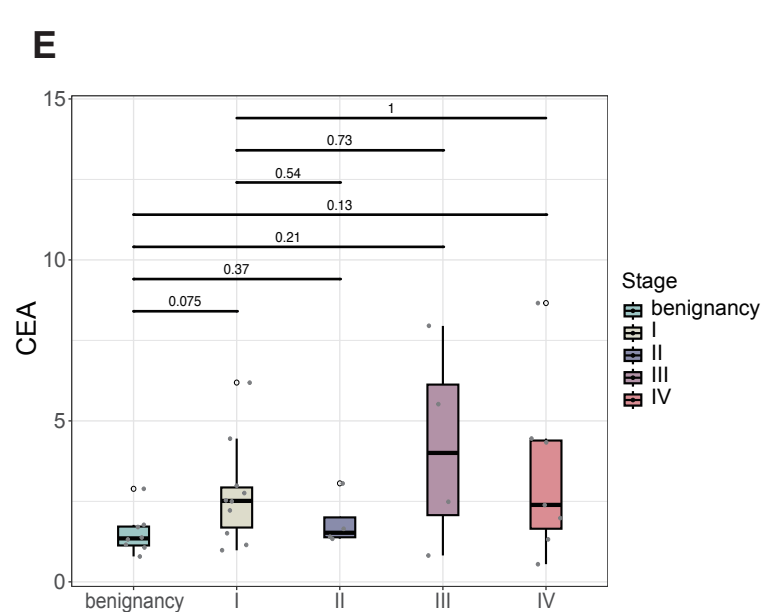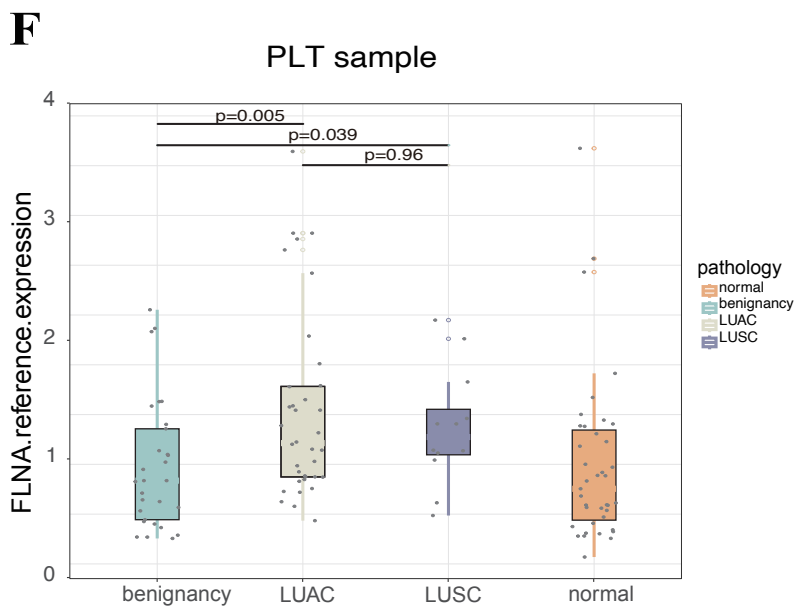

Supplement: Supplementary file 6 — Figure S5. Exploration of serum tumour marker levels in cohort 2. (A) Expression of CYFRA21‐1 and its comparison in patients with benign lung nodules, stage I, II, III, and IV NSCLC, presented as box plots. (B) Expression of NSE and its comparison in patients with benign lung nodules, stage I, II, III, and IV NSCLC, presented as box plots. (C) Expression of Pro.GRP and its comparison in patients with benign lung nodules, stage I, II, III, and IV NSCLC, presented as box plots. (D)Expression of TPS and its comparison in patients with benign lung nodules, stage I, II, III, and IV NSCLC, presented as box plots. (E) Expression of CEA and its comparison in patients with benign lung nodules, stage I, II, III, and IV NSCLC, presented as box plots. (F) Expression of FLNA and its comparison in healthy controls, benign lung nodules, LUSC, and LUAD patients, presented as box plots. [file JCMM-29-e70544-s011.pdf]

**A**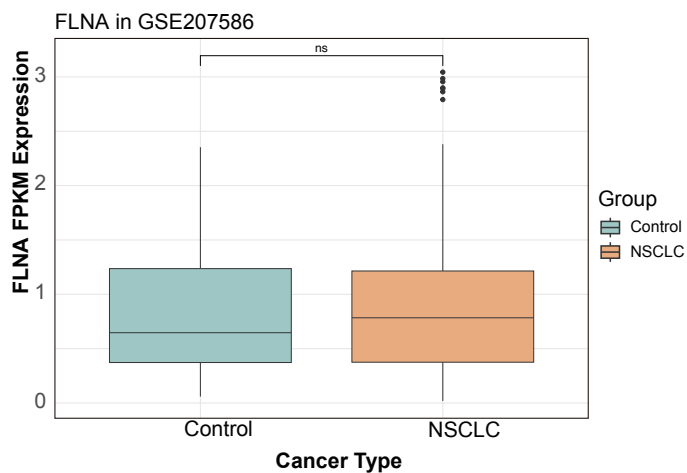**B**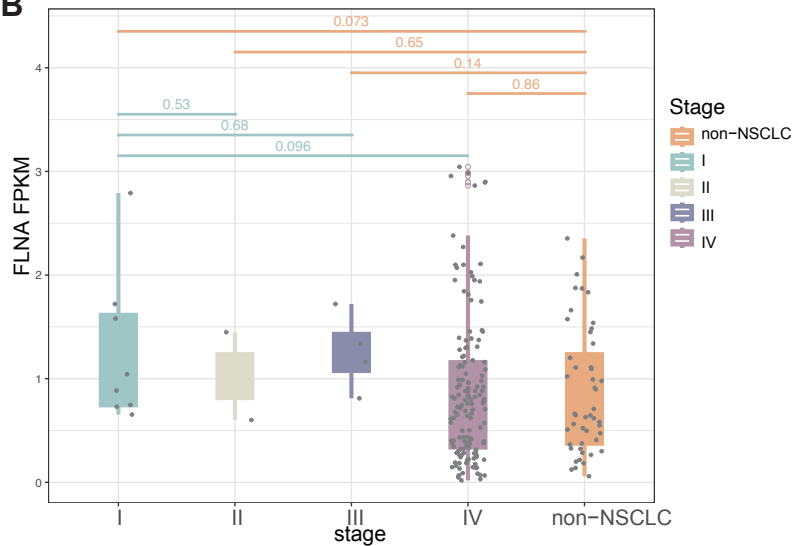**C**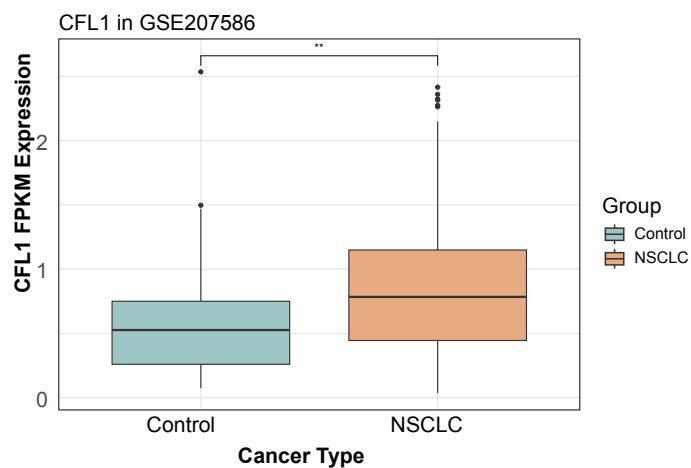**D**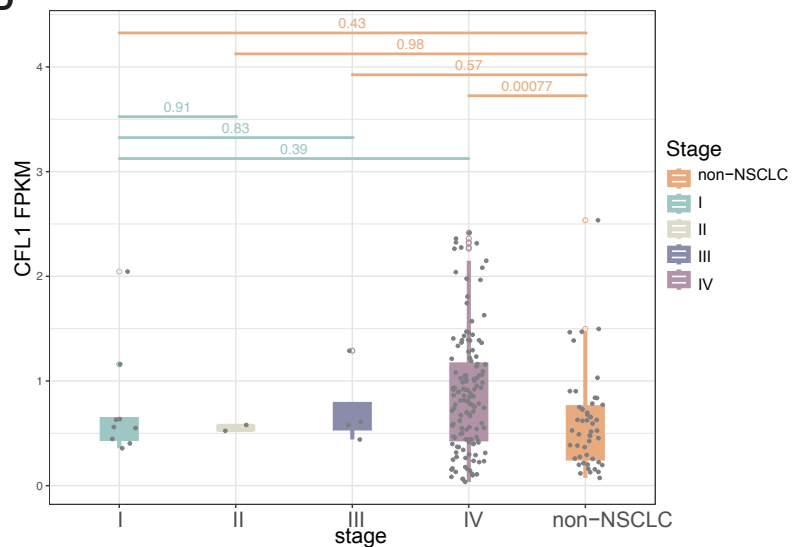**E**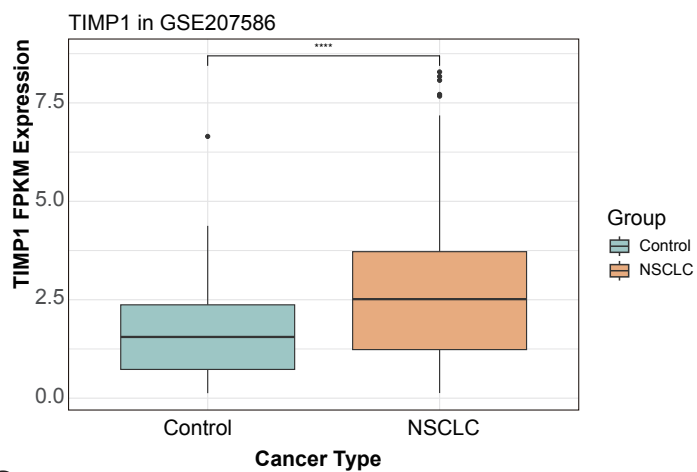**F**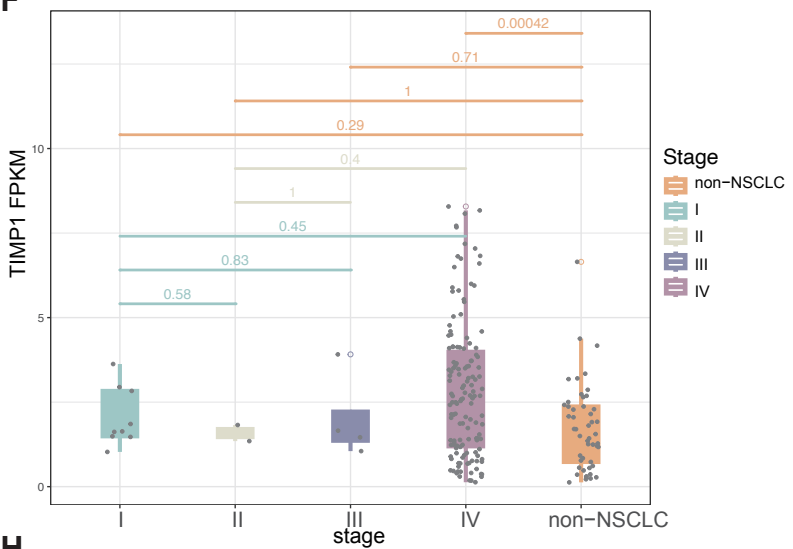**G**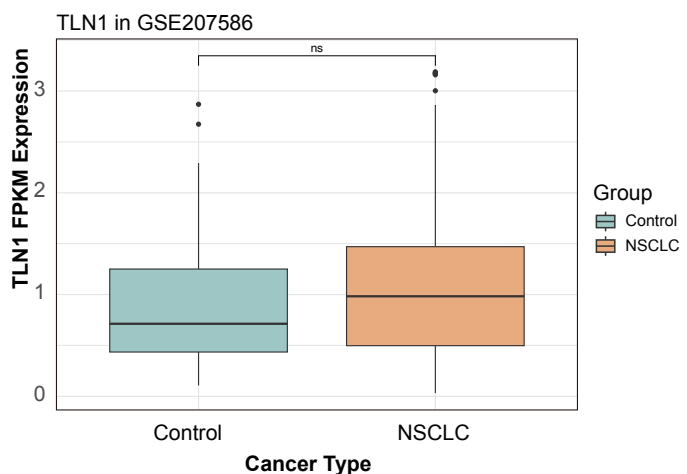**H**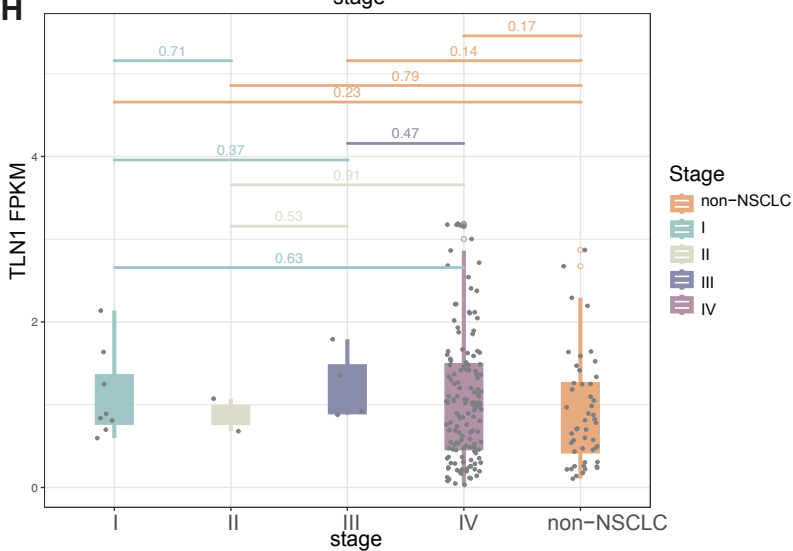

Supplement: Supplementary file 7 — Figure S6. Exploration of FLNA, TLN1, TIMP1, and CFL1 Gene Expression in GSE207586. (A) Bar plots showing the comparison of FPKM values for FLNA between NSCLC patients and controls in the GSE207586 dataset. (B) Bar plots showing the comparison of FPKM values for FLNA between different stage of NSCLC patients and controls in the GSE207586 dataset. (C) Bar plots showing the comparison of FPKM values for CFL1 between NSCLC patients and controls in the GSE207586 dataset. (D) Bar plots showing the comparison of FPKM values for CFL1 between different stage of NSCLC patients and controls in the GSE207586 dataset. (E) Bar plots showing the comparison of FPKM values for TIMP1 between NSCLC patients and controls in the GSE207586 dataset. (F) Bar plots showing the comparison of FPKM values for TIMP1 between different stage of NSCLC patients and controls in the GSE207586 dataset. (G) Bar plots showing the comparison of FPKM values for TLN1 between NSCLC patients and controls in the GSE207586 dataset. (H) Bar plots showing the comparison of FPKM values for TLN1 between different stage of NSCLC patients and controls in the GSE207586 dataset. [file JCMM-29-e70544-s001.pdf]

**A**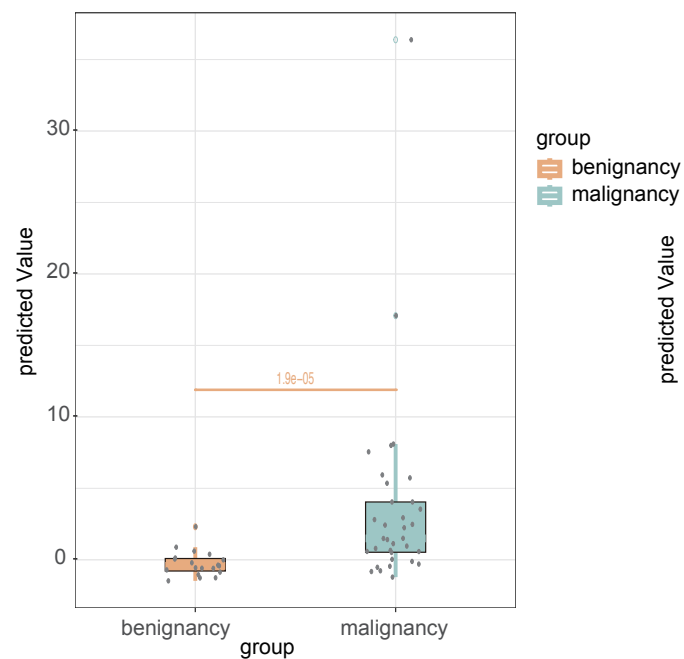**B**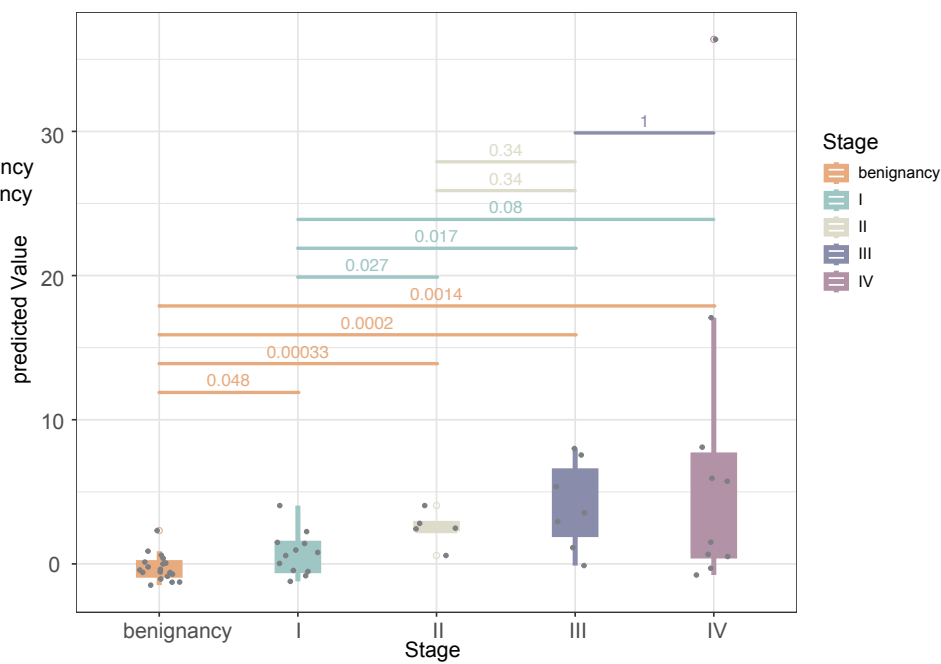**C**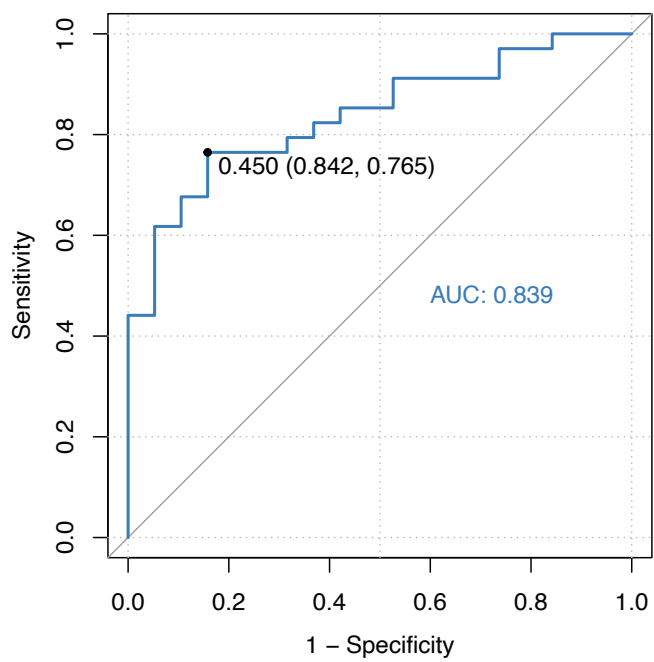**D**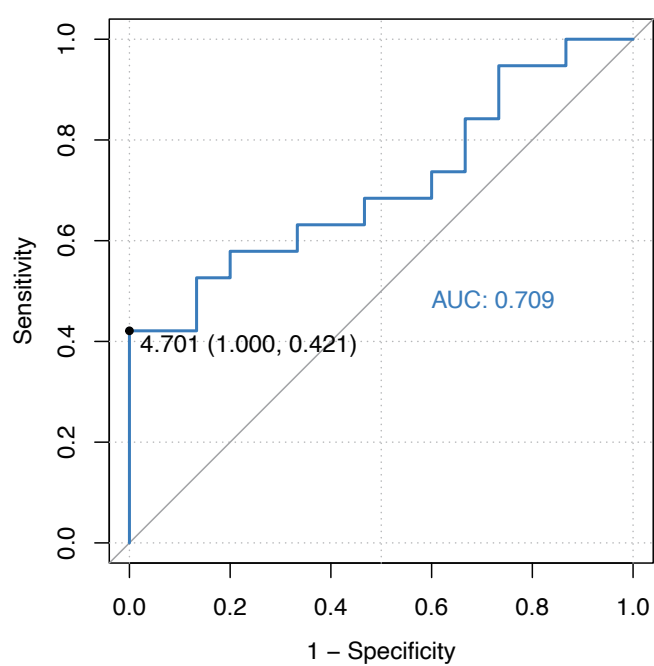

Supplement: Supplementary file 8 — Figure S7. The dianostic performance of the combined model constructing with FLNA and CYFRA 21‐1. (A) Bar plots showing the comparison of predicted values between NSCLC patients and benignant patients in the cohort2. (B) Expression of predicted values and its comparison in patients with benign lung nodules, and NSCLC, presented as box plots. (C) ROC curves for predicted values in differentiating NSCLC from benignancy based on cohort2. (D) ROC curves for predicted values in differentiating metastasis NSCLC from non‐metastasis NSCLC based on cohort2. [file JCMM-29-e70544-s007.pdf]
